# Supplementary material for: Probabilistic day-ahead forecasting of system-level renewable energy and electricity demand
Source: Nat Commun. 2026 Feb 28;17:3307. doi: 10.1038/s41467-026-69015-w (PMC13065771; doi:10.1038/s41467-026-69015-w)
Supplement: Supplementary file 1 — Supplementary Information [file 41467_2026_69015_MOESM1_ESM.pdf]

Supplementary Information: Probabilistic Day-Ahead Forecasting of System-Level Renewable Energy and Electricity Demand

Guillermo Terrén-Serrano[1,2,*], Ranjit Deshmukh[1,2,3], Manel Martínez-Ramón[4]

[1] *Environmental Studies, University of California Santa Barbara, Santa Barbara, CA, USA*
[2] *Environmental Markets Lab (emLab), University of California Santa Barbara, Santa Barbara, CA, USA*
[3] *Bren School of Environmental Sciences and Management, University of California Santa Barbara, Santa Barbara, CA, USA*
[4] *Department of Electrical and Computer Engineering, University of New Mexico, Albuquerque, NM, USA*

[*] Corresponding author details: guillermoterren@ucsb.edu

Supplementary Items

Supplementary Figures 3
Supplementary Figure 1 . . . 3
Supplementary Figure 2 . . . 4
Supplementary Figure 3 . . . 5
Supplementary Figure 4 . . . 6
Supplementary Figure 5 . . . 6
Supplementary Figure 6 . . . 7
Supplementary Figure 7 . . . 8
Supplementary Figure 8 . . . 9
Supplementary Figure 9 . . . 10
Supplementary Figure 10 . . . 11
Supplementary Figure 11 . . . 12
Supplementary Figure 12 . . . 12
Supplementary Figure 13 . . . 13
Supplementary Figure 14 . . . 13
Supplementary Figure 15 . . . 14
Supplementary Figure 16 . . . 14
Supplementary Figure 17 . . . 15
Supplementary Figure 18 . . . 15
Supplementary Figure 19 . . . 16

Supplementary Tables 17
Supplementary Table 1 . . . 17
Supplementary Table 2 . . . 17
Supplementary Table 3 . . . 18
Supplementary Table 4 . . . 18

Supplementary Notes 19
Supplementary Note 1 . . . 19
Supplementary Note 2 . . . 20
Supplementary Note 3 . . . 20
Supplementary Note 4 . . . 23
Supplementary Note 5 . . . 23
Supplementary Note 6 . . . 24

Supplementary References 27

Supplementary Figures

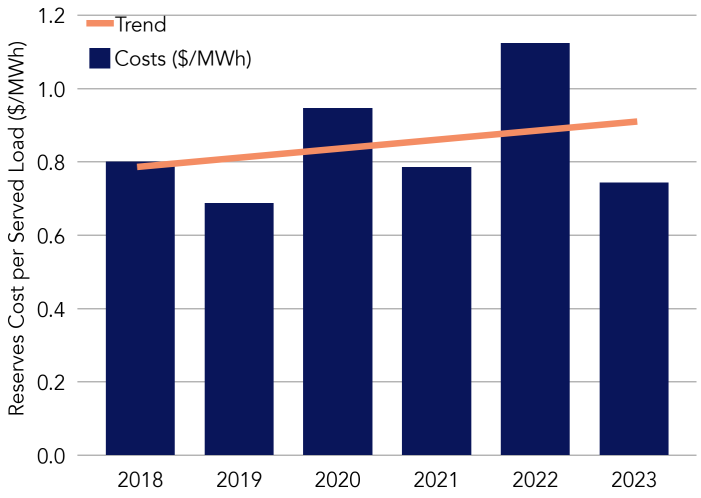

Supplementary Figure 1: The additional costs of the operational reserves in the auxiliary services market operated by CAISO, have been steadily increasing from 2018 [1, 2]. The blue bars show the marginal cost of the operational reserves per each unit of served load from 2018 to 2023 (USD/MWh). The orange line shows the linear trend fitted with the yearly average cost of operational reserves per each unit of served load as observations.

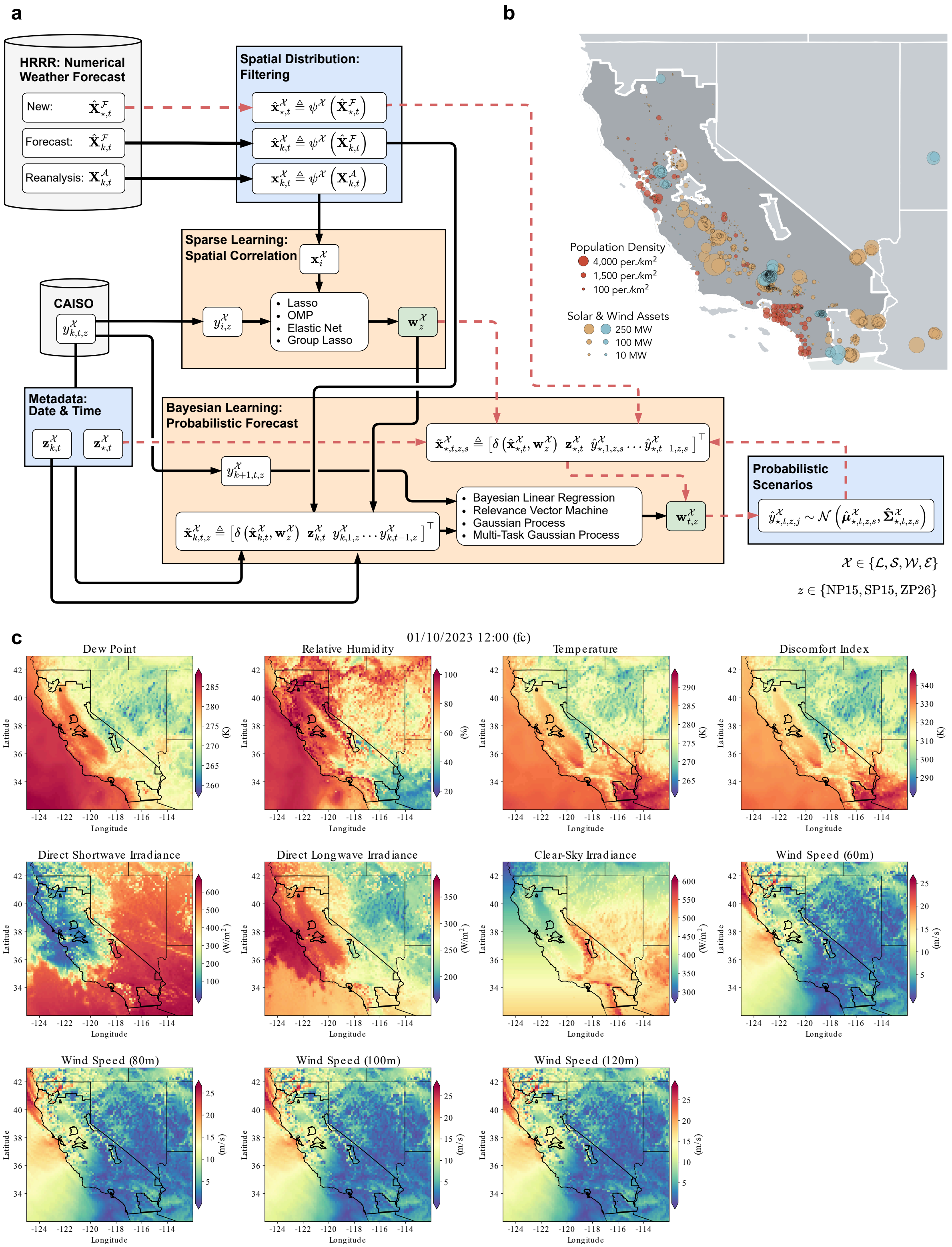

Supplementary Figure 2: (a) Workflow followed in the proposed method. The spatial points in the numerical weather forecast are filtered first using the spatial masks. Later, the actual weather features are used in a sparse learning model to discover the points in space more correlated with the electricity demand and solar and wind generation (from CAISO). Then, the forecasted weather features (from the previously identified points only) are used in a Bayesian model to predict electricity demand and solar and wind generation. Lines in black depict the training pipeline, and dashed red lines depict the testing pipeline. (b) Population density, and solar and wind power plant ($\geq$ 1MW) location. (c) Forecasted weather features obtained from the HRRR-NWF on 01/09/2023 at 4 pm for the next day at 12 pm. Map data © Esri; sources: California Energy Commission and Natural Earth [3, 4].

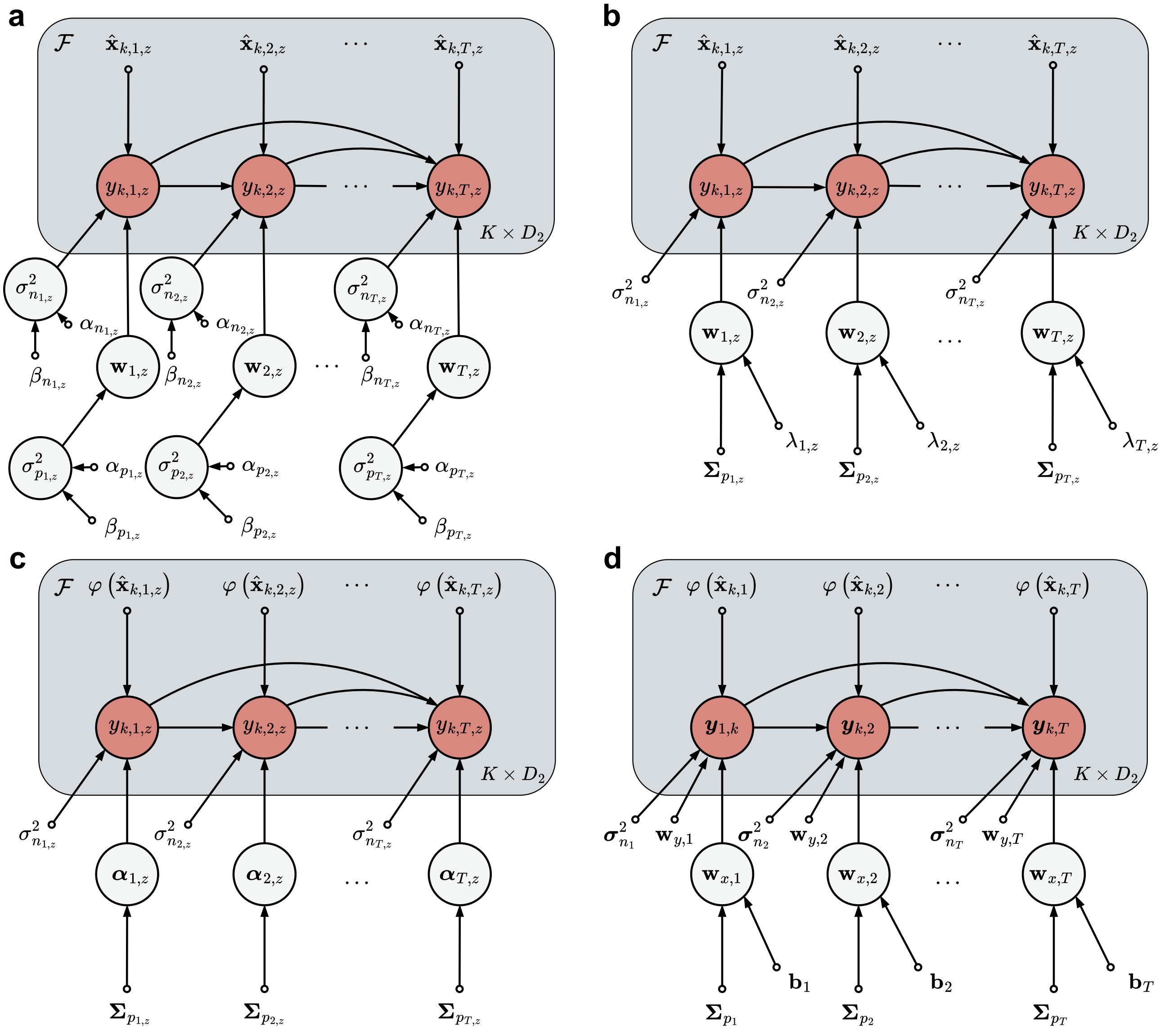

Supplementary Figure 3: Graphical models of the Bayesian methods implemented in this research: (a) Bayesian Linear Regression (BLR), (b) Relevance Vector Machine (RVM), (c) Gaussian Process for Regression (GPR), and (d) Multi-Task GPR (MTGPR). The gray rectangles represent the weather forecast dataset ($\mathcal{F}$). The circles are probabilistic distributions placed on the variable. The letters on the gray circles are latent variables, and the ones in the red circles are observable. The directed edges indicate the dependence between variables. Variables outside the circles and the rectangle are parameters of the probability distribution that require inference.

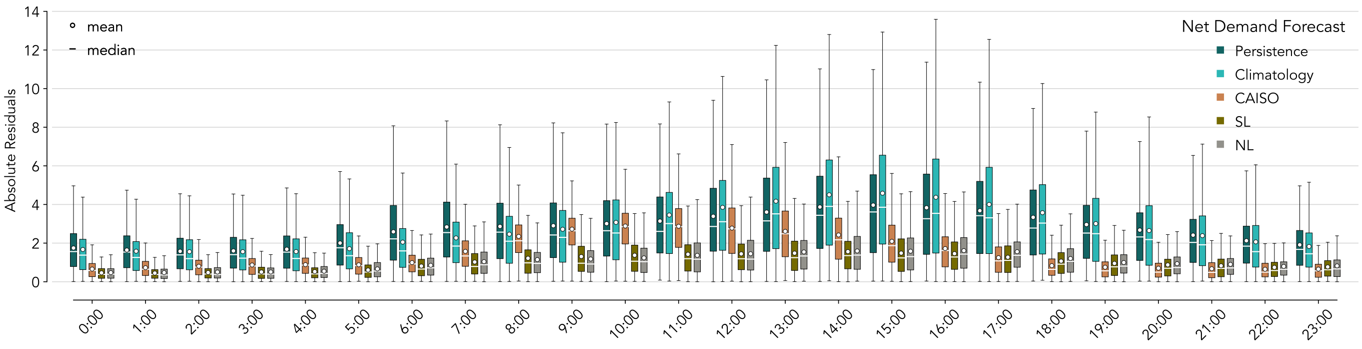

Supplementary Figure 4: Hourly distributions of absolute residual errors for the net-demand day-ahead system-level forecast. Boxplots show hourly absolute residuals for baseline day-ahead forecasts: persistence (light green), climatology (green), and the CAISO forecast (orange). Because the proposed day-ahead forecasts are probabilistic, residuals are computed using the predictive mean. Residuals from system-level (SL) models are shown in light brown, while those from node-level (NL) models are shown in dark green.

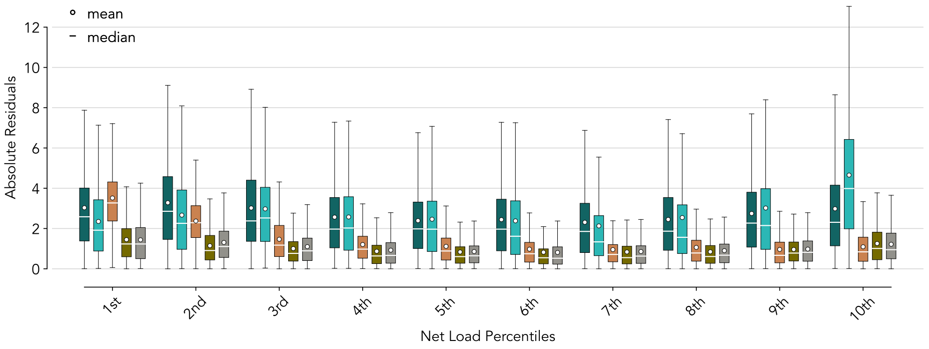

Supplementary Figure 5: Distributions of hourly absolute residuals grouped by net-demand magnitude deciles. Boxplots show absolute residuals grouped by net-demand deciles for baseline day-ahead forecasts: persistence (light green), climatology (green), and the CAISO forecast (orange). Residuals from system-level (SL) models are shown in light brown, while those from node-level (NL) models are shown in dark green. The underlying hourly absolute residuals are identical to those shown in supplementary figure 4 and are regrouped here by net-demand magnitude.

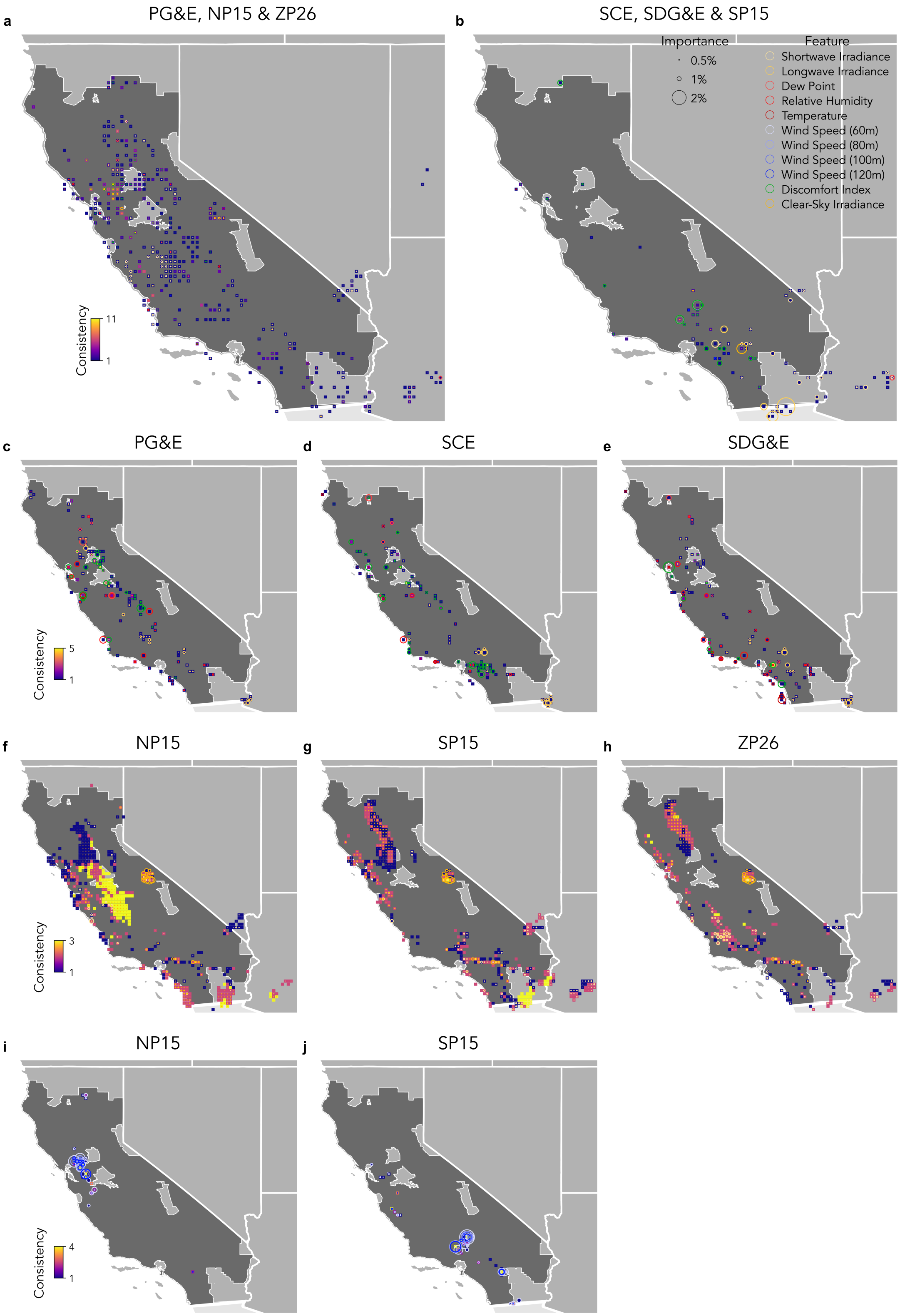

Supplementary Figure 6: Features maps showing the weather features selected by the sparse models in the independent forecasts of electricity demand (c, d, e), solar generation (f, g, h), and wind generation (i, j). The feature maps for the joint forecast of multiple energy features per node are panels (a) and (b). The consistency $\sum_t \mathbb{I}(\boldsymbol{\beta}_t > 0)$ of selected features (square marker) has a color gradient (brighter, more consistent). The importance of the weights $\frac{\boldsymbol{\beta}_t}{\|\boldsymbol{\beta}_t\|_2}$ of each feature is the diameter of the circular marker. The circular marker color represents the feature group: solar radiation (orange), wind speed (blue), atmospheric (red), and comfort index (green). Map data © Esri; sources: California Energy Commission and Natural Earth [3, 4].

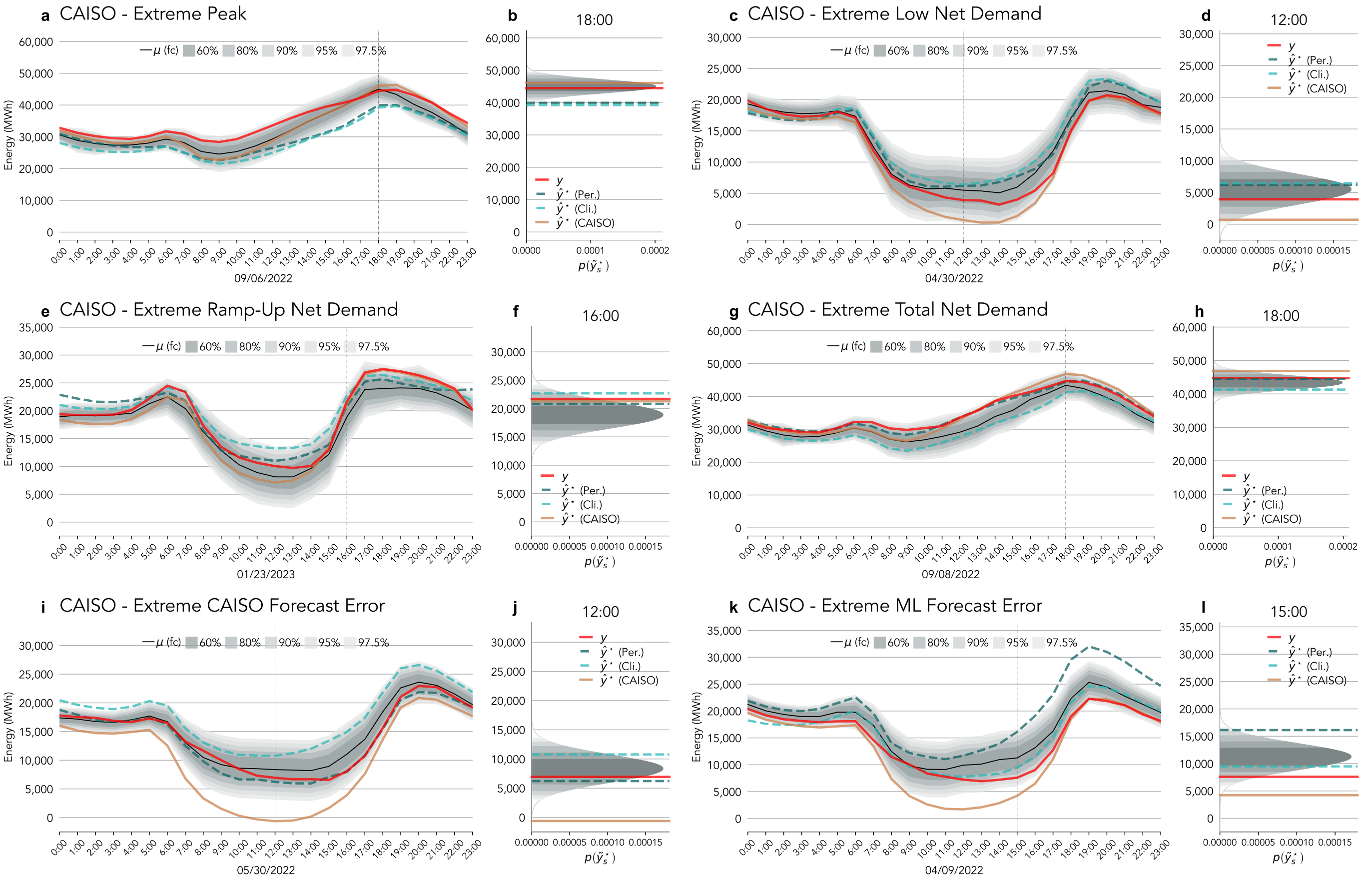

Supplementary Figure 7: Probabilistic day-ahead net demand forecast on extreme events at the system level (CAISO). The events considered extreme are high peak (a, b), low net demand (c, d), ramp-up (e, f), high net demand (g, h) aggregated pointwise error in the forecast provided by CAISO (i, j), and aggregated pointwise error in the predictive mean from the proposed probabilistic forecast(k, l). The predictive mean from the probabilistic forecast $\boldsymbol{\mu}^{\star}$ is black. The probabilistic forecasts are compared to the actual $y^{\star}$ (red), and the baselines $\hat{\mathbf{y}}^{\star}$ Persistence (dashed light green), Climatology (dashed dark green), and CAISO (orange). The represented predictive intervals (60%, 80%, 90%, 95%, and 97.5%) have a color gradient that goes from dark (60%) to light gray (97.5%). The dashed marks the hour of the detail in (c, f, i).

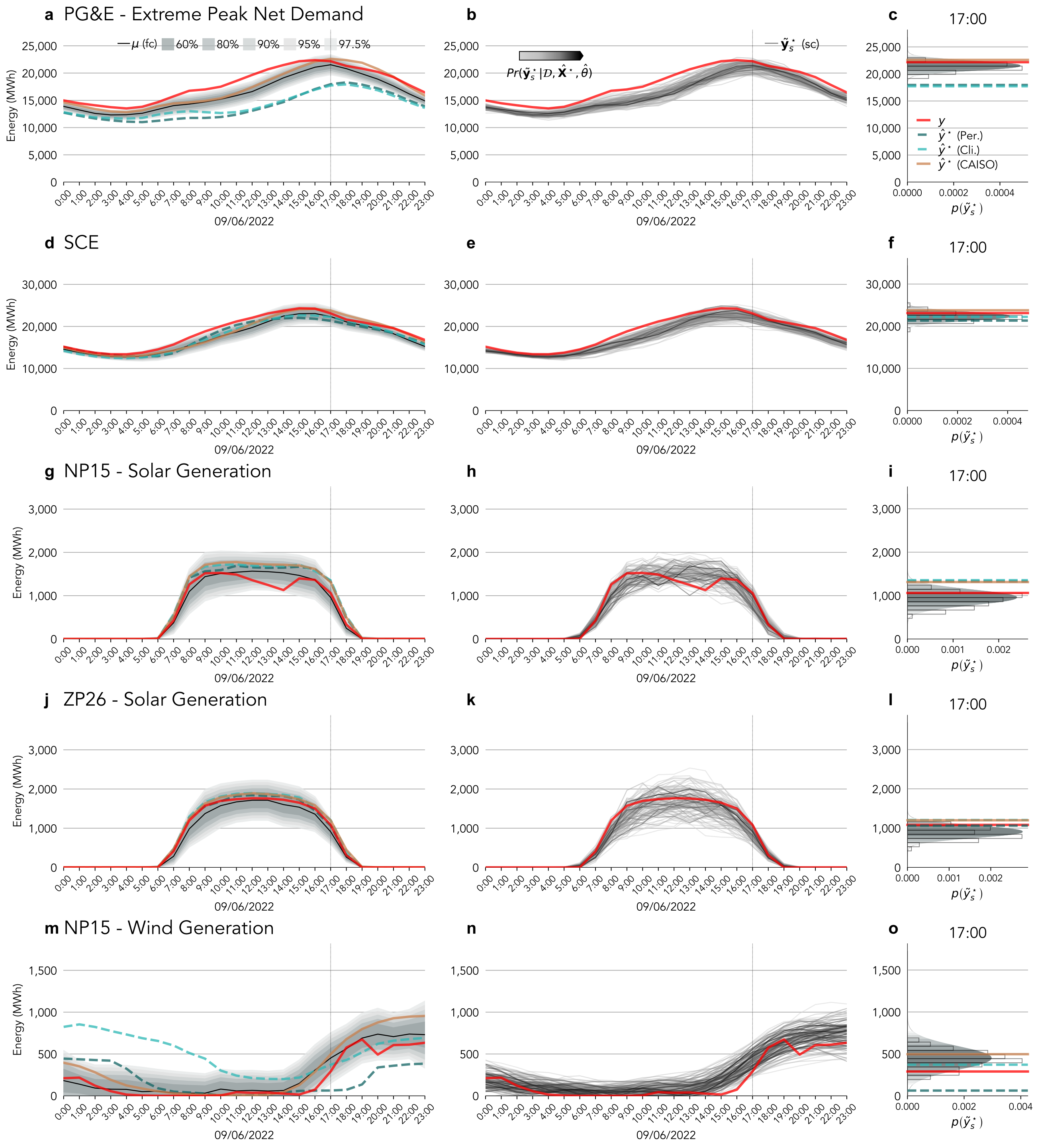

Supplementary Figure 8: Independent probabilistic day-ahead forecasts during the extreme peak demand event on Sep. 6, 2022. Probabilistic day-ahead solar generation forecast for PG&E (a, b, and c) and SCE (d, e, and f). Probabilistic day-ahead solar generation forecast at NP15 (g, h, and i) and ZP26 (j, k, and l). Probabilistic day-ahead wind generation forecast at NP15 (m, n, and o). (a, d, g, j, and m) Probabilistic forecast predictive mean $\boldsymbol{\mu}^\star$ compared to the actual $y^\star$, and the baselines $\hat{\mathbf{y}}^\star$ (persistence, climatology, and CAISO). The represented predictive intervals (60%, 80%, 90%, 95%, and 97.5%) have a color gradient from dark (60%) to light gray (97.5%). (b, e, h, k, and n) dashed lines mark detailed hours in (c, f, i, l, and o). The color gradient in the predictive scenarios represents the probability $\Pr(\tilde{\mathbf{y}}^\star_s \mid \mathcal{D}, \hat{\Theta}, \hat{\mathbf{X}}^\star)$ of the sth scenario $(\hat{y}^\star_s)$ of electricity demand $(\mathcal{L})$, solar $(\mathcal{S})$ or wind $(\mathcal{W})$ generation (darker means higher probability).

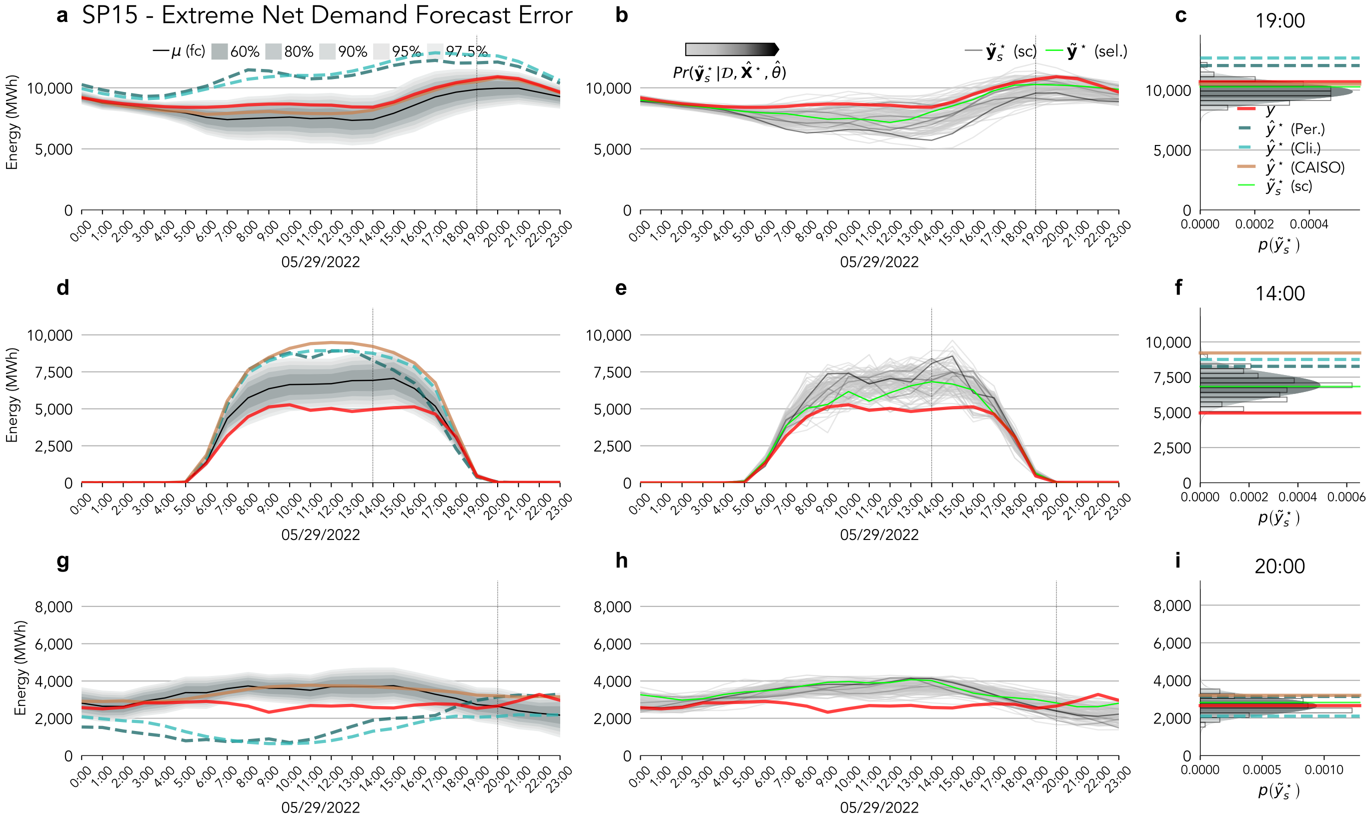

Supplementary Figure 9: (a, d, g) Joint forecast predictive mean $\boldsymbol{\mu}^\star$, actual $\mathbf{y}^\star$ and the baseline forecasts $\hat{\mathbf{y}}^\star$ (persistence, climatology, and CAISO) of SCE electricity demand (a), solar (d), and wind generation (g) at SP15. The lines are joint scenarios drawn from the predictive density function (the gray color intensity represents their probability). The highlighted scenario is a joint draw of electricity demand (b), solar generation (e), and wind generation (h). Density function details (c, f, i) are of the hours (marked by vertical dashed lines) with the largest error between the predictive mean $\mu_t^\star$ and the actual $y_t^\star$.

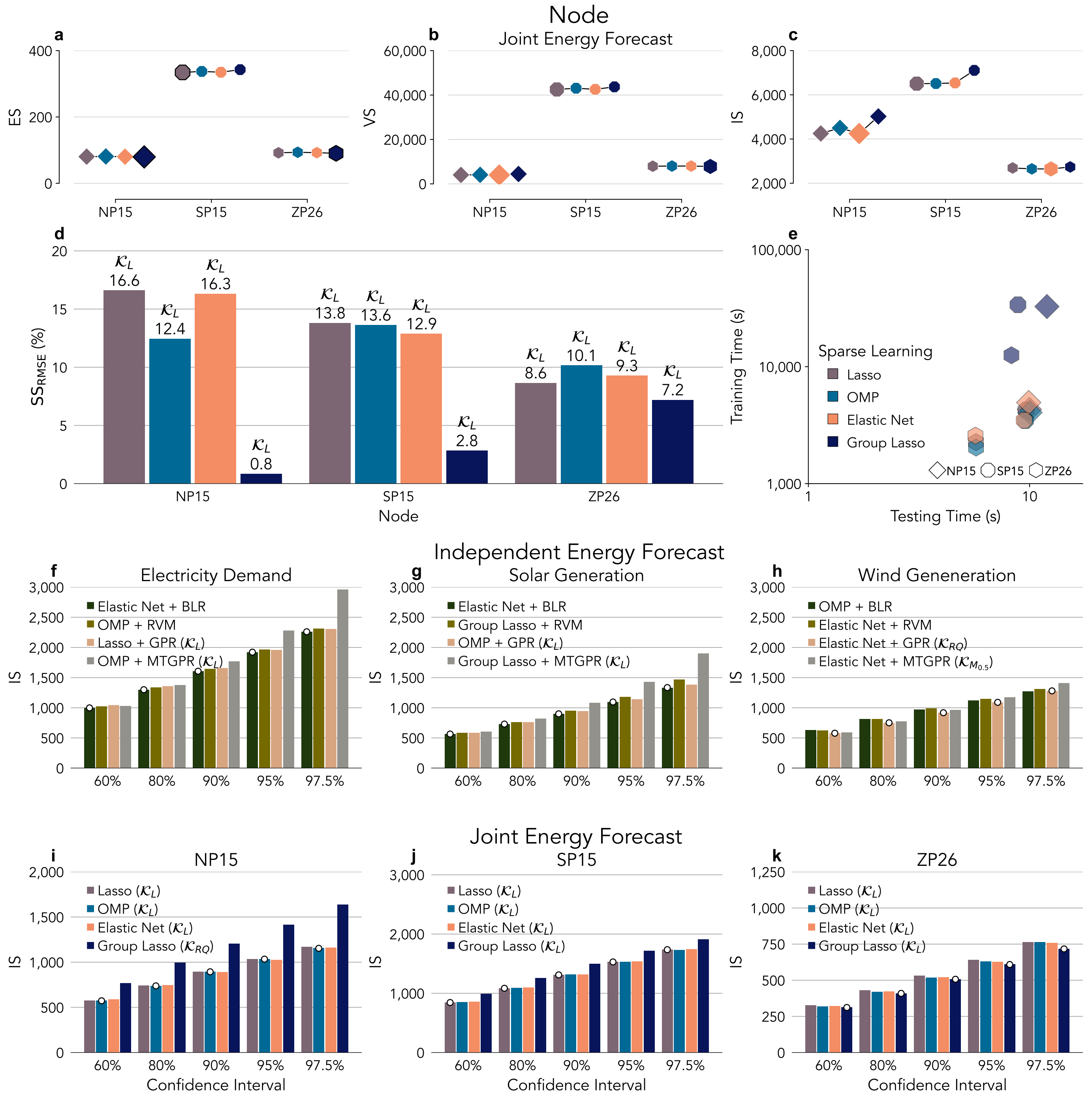

Supplementary Figure 10: The figures (a-e) show joint day-ahead energy forecast at the node level model selection based on validation Energy Score (ES). The colors represent the different sparse methods: Lasso (gray), OMP (blue), Elastic Net (orange), and Group Lasso (dark blue). The markers represent the node NP15 (rhombus), SP15 (circle), and ZP26 (pentagon). Lower ES, IS $VS^{0.5}$ and computing time is preferable, while higher SS_{RMSE} is preferable. The scores achieved by the sparse and Bayesian model combinations, (a) ES, (b) Variogram Score ($VS^{0.5}$), (c) IS, (d) SS_{RMSE}, and (e) computing time. The figures (f-h) show the independent forecast model selection for the electricity demand (f), solar (g), and wind (h) generation based on the validation Interval Score (IS). The colors represent the Bayesian method: BLR (dark green), RVM (green), GPR (brown), and MTGPR (gray). The figures (i-k) show a joint energy forecast for the NP15 (i), SP15 (j), and ZP26 (k) nodes based on validation IS.

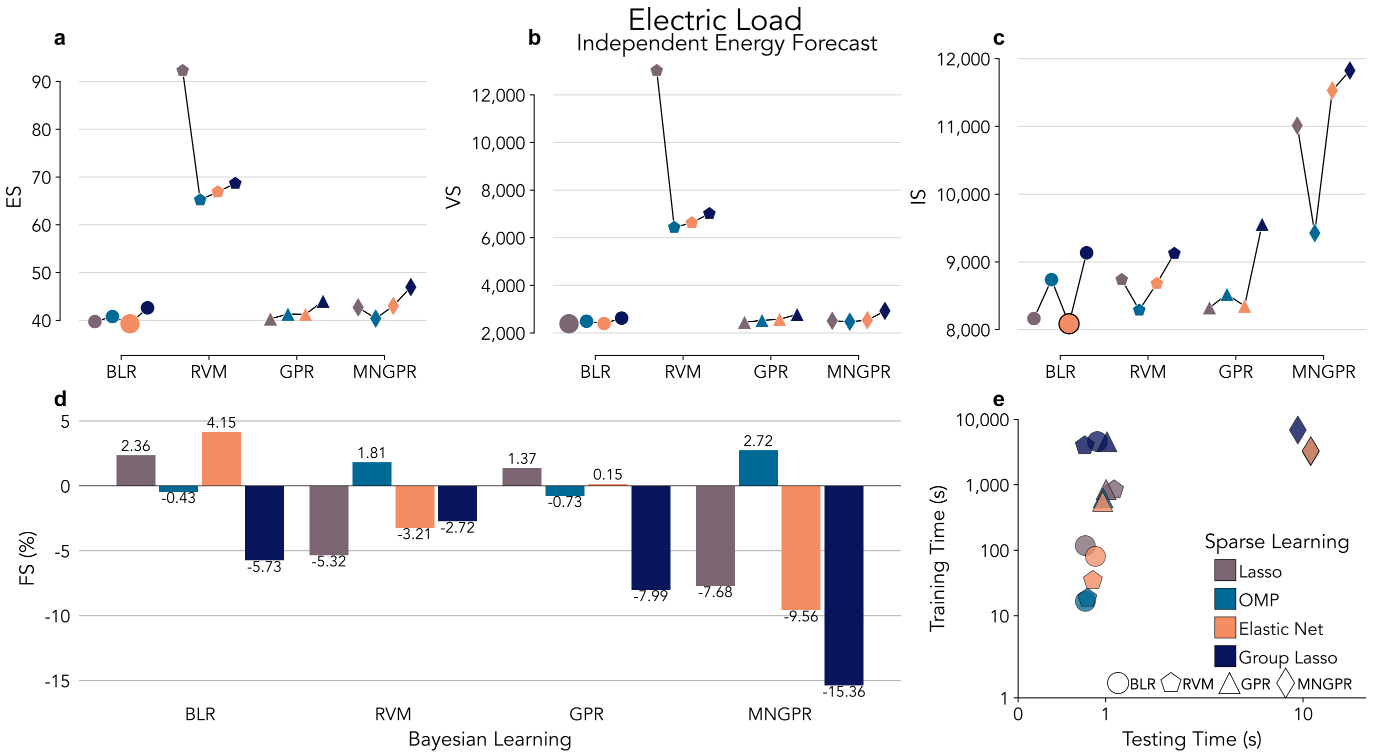

Supplementary Figure 11: Independent electric load day-ahead probabilistic forecast model section by lower Ignorance Score (IS). (a) Energy Score (ES), (b) Variogram Score with $p = 0.5$ ($\mathrm{VS}^{0.5}$), and (c) IS. (d) Skill Score based on RMSE ($\mathrm{SS}_{\mathrm{RMSE}}$) relative to CAISO's forecast, and (e) training and testing computing time.

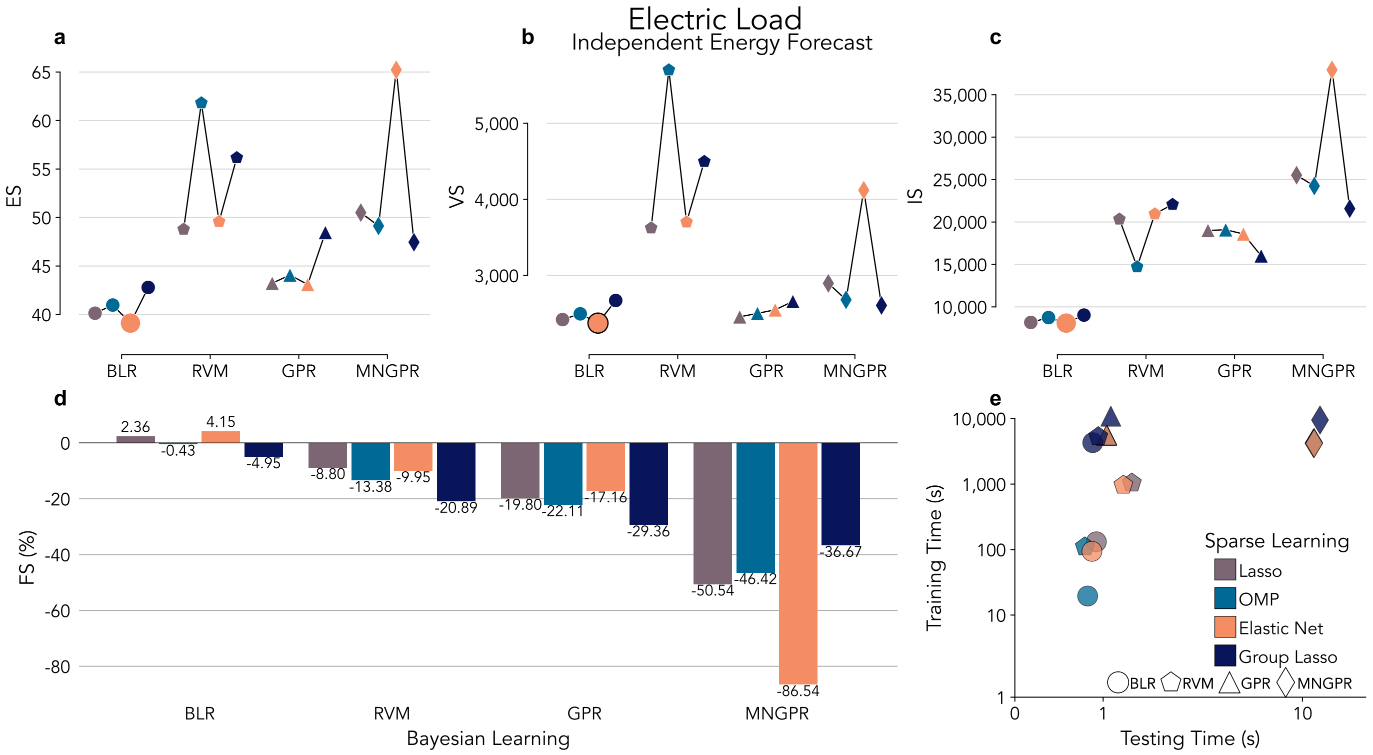

Supplementary Figure 12: Independent electric load day-ahead probabilistic forecast model section by lower Variogram Score with $p = 0.5$ ($\mathrm{VS}^{p=0.5}$). (a) Energy Score (ES), (b) $\mathrm{VS}^{0.5}$, and (c) Ignorance Score (IS). (d) Skill Score based on RMSE ($\mathrm{SS}_{\mathrm{RMSE}}$) relative to CAISO's forecast, and (e) training and testing computing time.

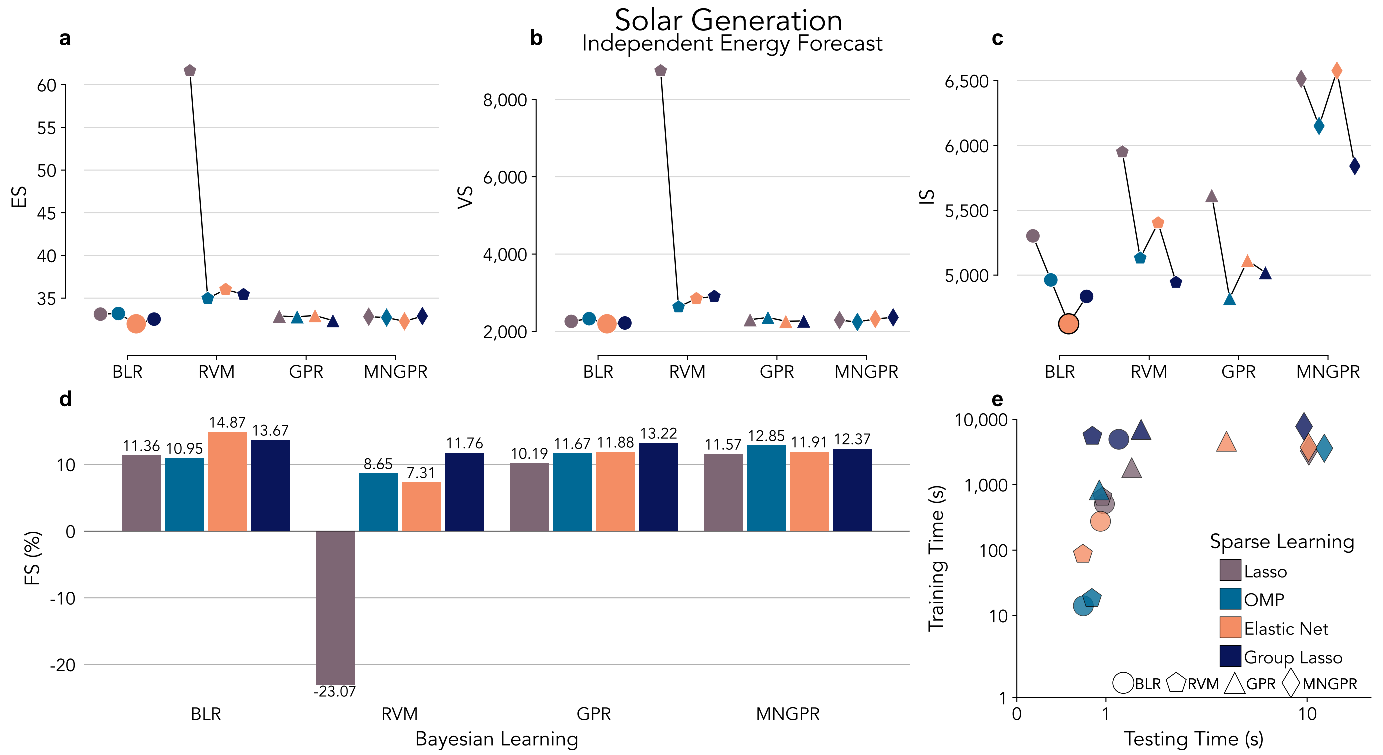

Supplementary Figure 13: Independent solar energy generation day-ahead probabilistic forecast model section by lower Ignorance Score (IS). (a) Energy Score (ES), (b) Variogram Score with $p = 0.5$ ($VS^{0.5}$), and (c) IS. (d) Skill Score based on RMSE (SS_{RMSE}) relative to CAISO's forecast, and (e) training and testing computing time.

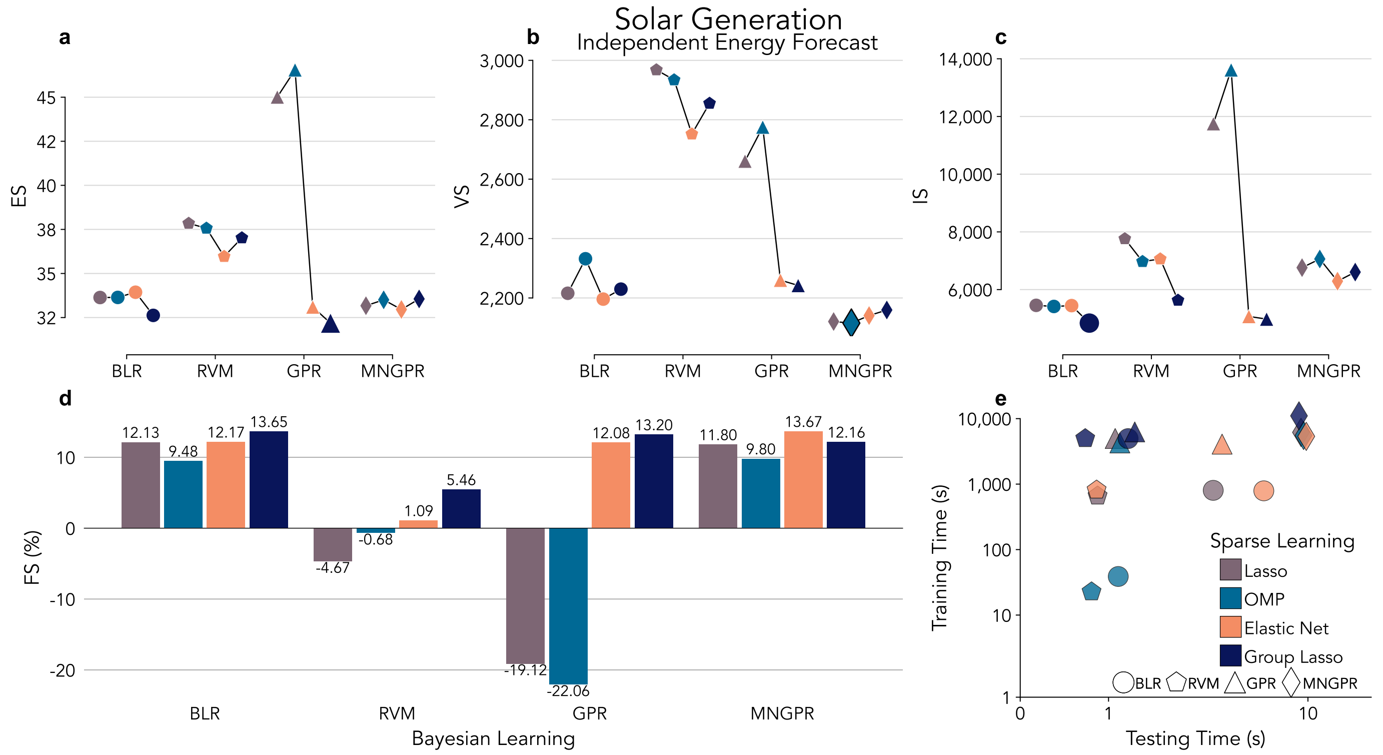

Supplementary Figure 14: Independent solar energy generation day-ahead probabilistic forecast model section by lower Variogram Score with $p = 0.5$ ($VS^{0.5}$). (a) Energy Score (ES), (b) $VS^{0.5}$, and (c) Ignorance Score (IS). (d) Skill Score based on RMSE (SS_{RMSE}) relative to CAISO's forecast, and (e) training and testing computing time.

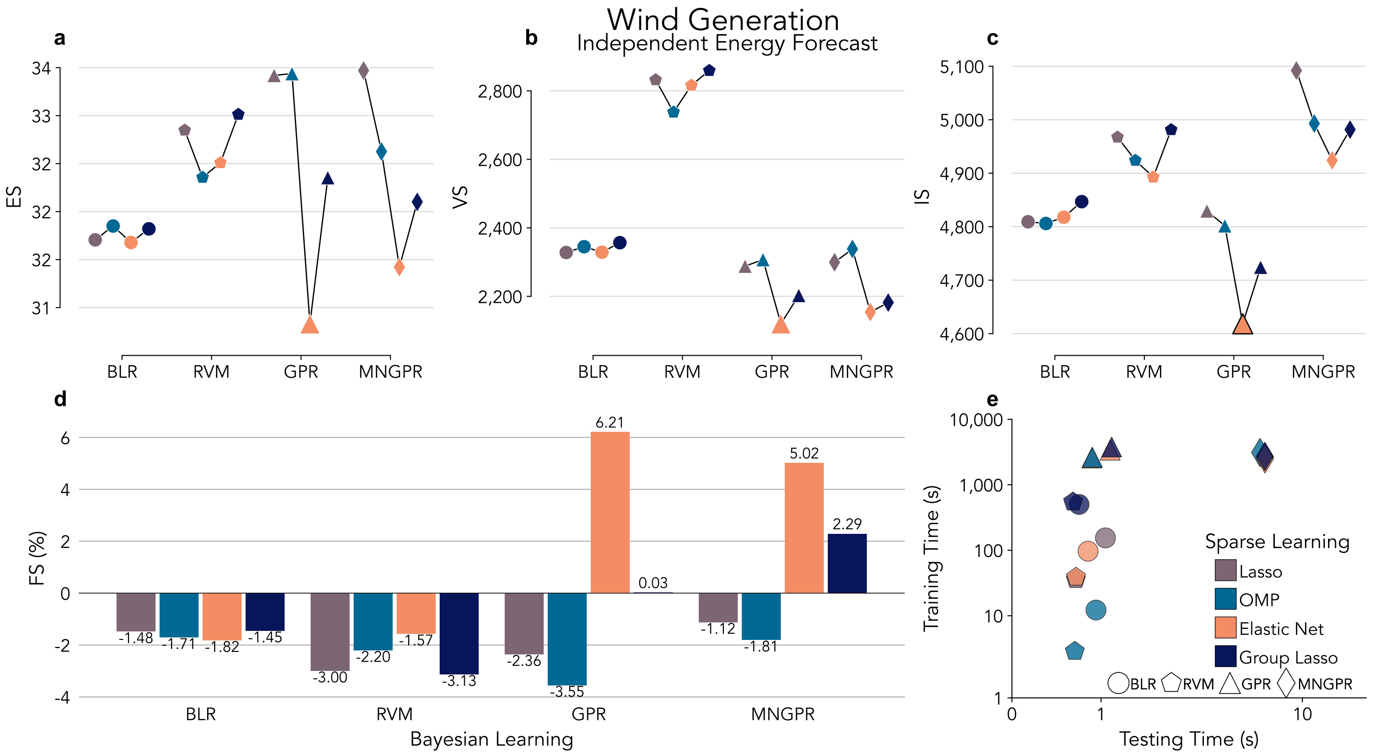

Supplementary Figure 15: Independent wind energy generation day-ahead probabilistic forecast model section by lower Ignorance Score (IS). (a) Energy Score (ES), (b) Variogram Score with $p = 0.5$ ($VS^{0.5}$), and (c) IS. (d) Skill Score based on RMSE (SS_{RMSE}) relative to CAISO's forecast, and (e) training and testing computing time.

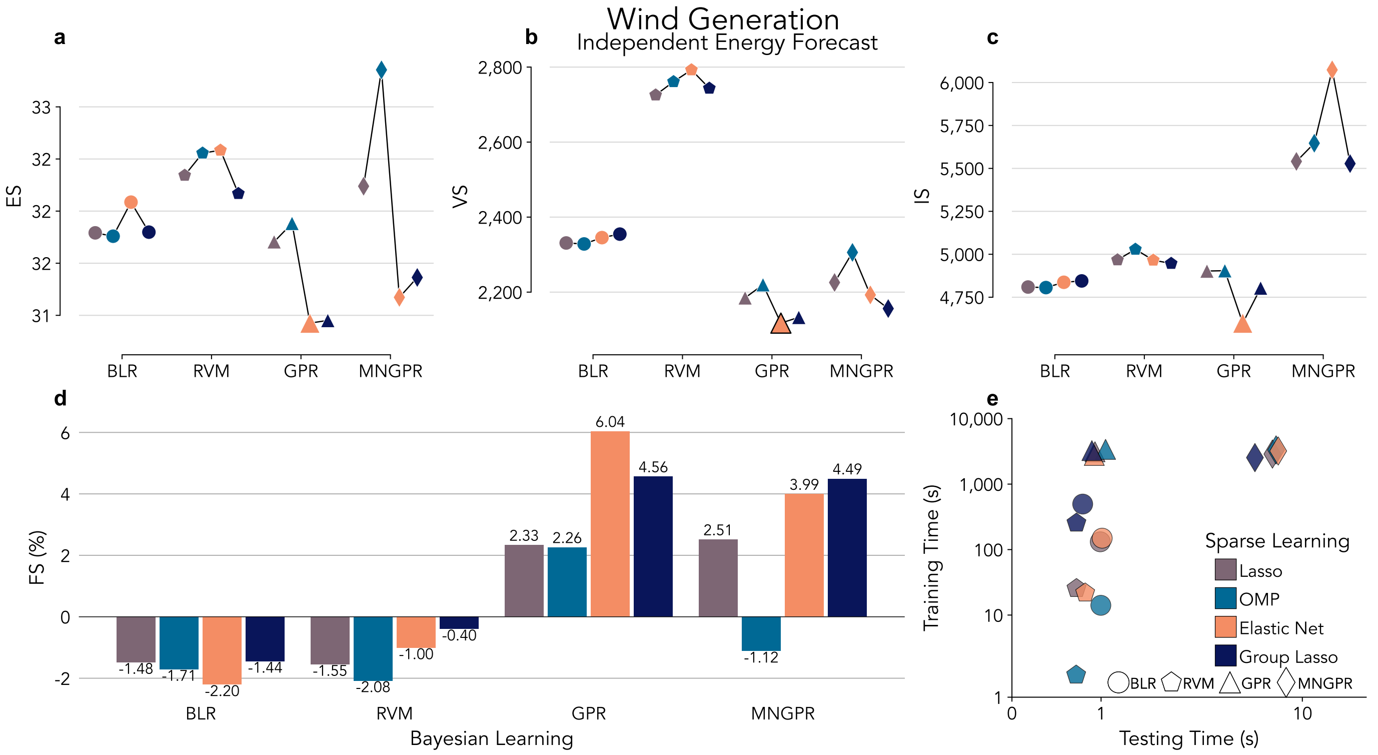

Supplementary Figure 16: Independent wind energy generation day-ahead probabilistic forecast model section by lower Variogram Score with $p = 0.5$ ($VS^{0.5}$). (a) Energy Score (ES), (b) $VS^{0.5}$, and (c) Ignorance Score (IS). (d) Skill Score based on RMSE (SS_{RMSE}) relative to CAISO's forecast, and (e) training and testing computing time.

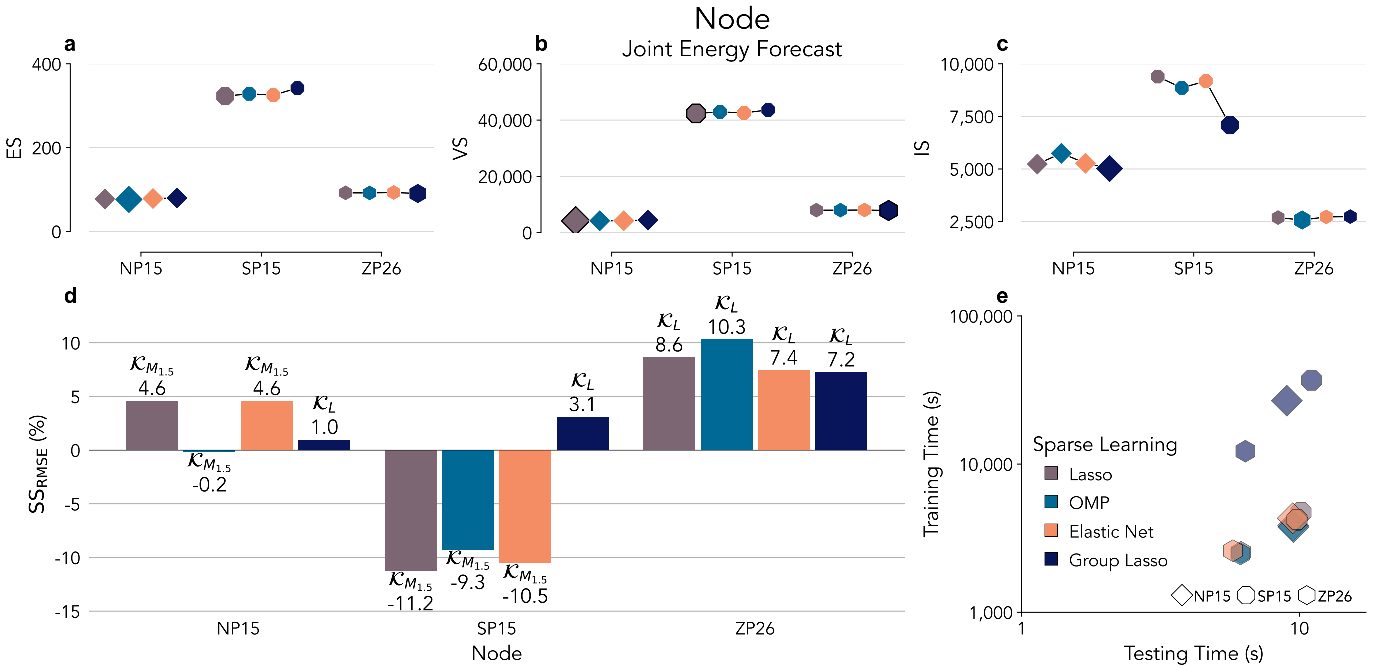

Supplementary Figure 17: Joint multi-resource day-ahead probabilistic forecast model section by lower Energy Score (ES). (a) ES, (b) Variogram Score with $p = 0.5$ ($VS^{0.5}$), and (c) IS. (d) Skill Score based on RMSE (SS_{RMSE}) relative to CAISO's forecast, and (e) training and testing computing time.

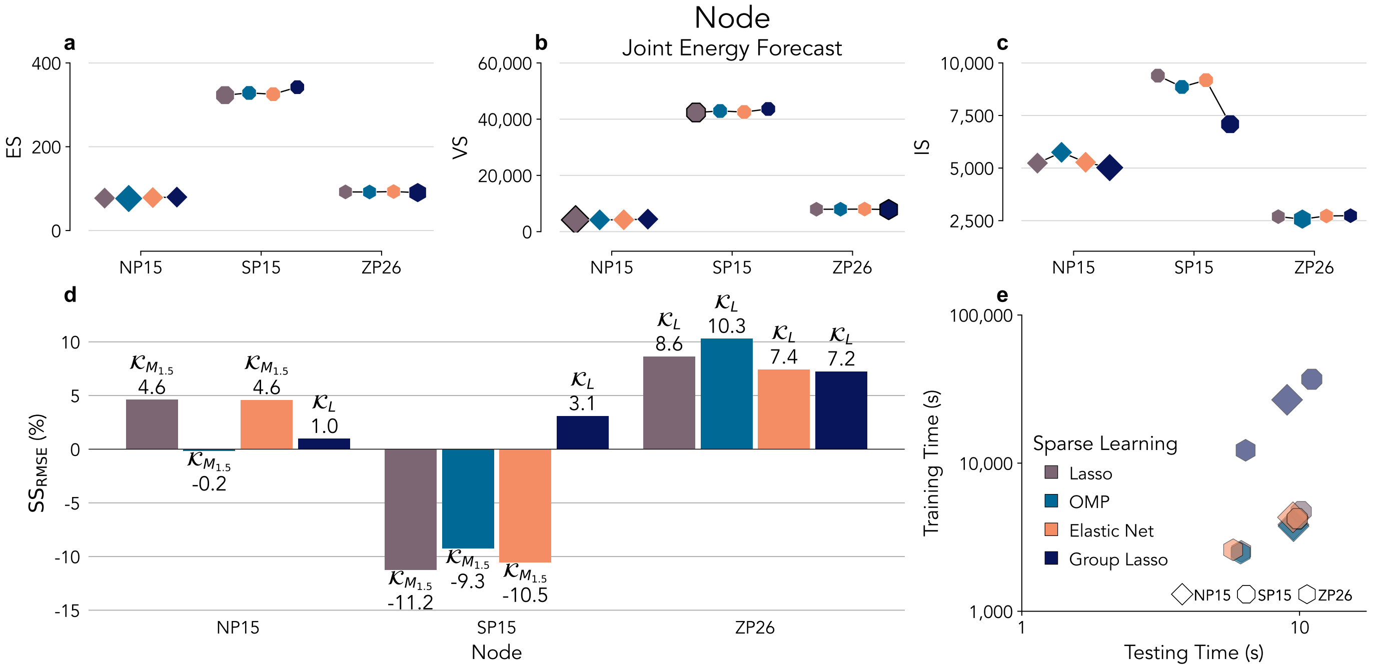

Supplementary Figure 18: Joint multi-resource day-ahead probabilistic forecast model section by lower Variogram Score with $p = 0.5$ ($VS^{0.5}$). (a) Energy Score (ES), (b) $VS^{0.5}$, and (c) Ignorance Score (IS). (d) Skill Score based on RMSE (SS_{RMSE}) relative to CAISO's forecast, and (e) training and testing computing time.

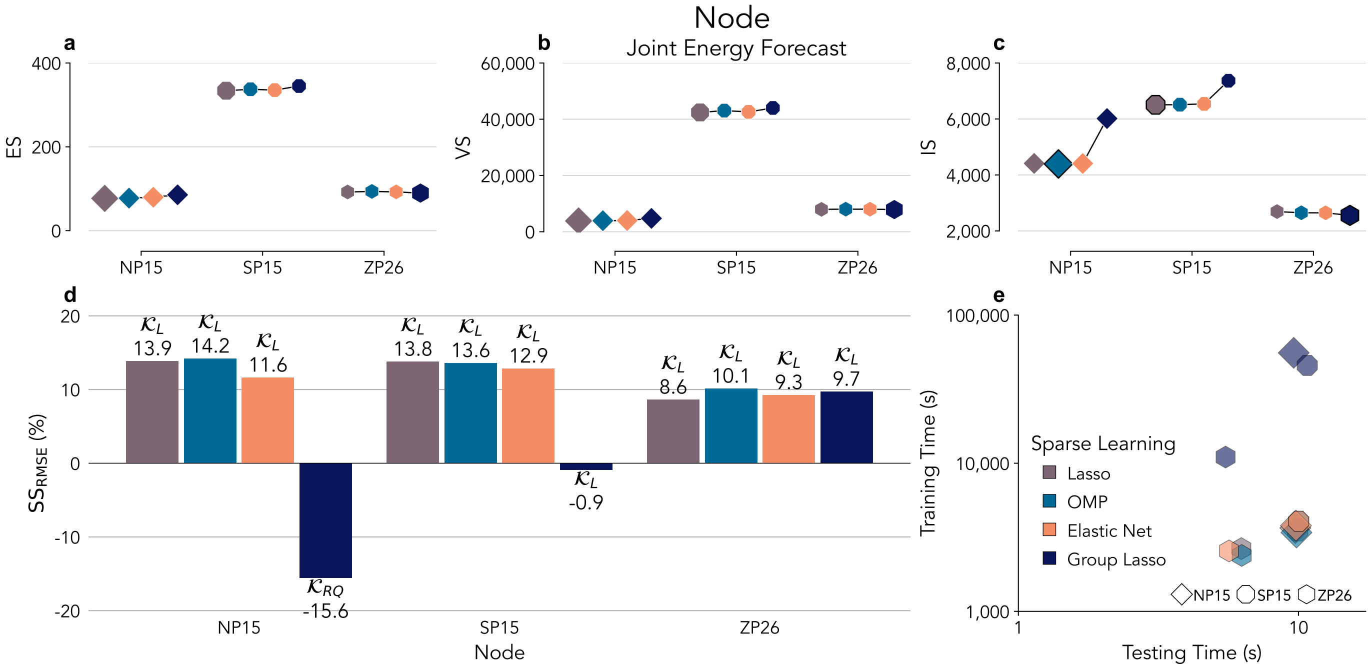

Supplementary Figure 19: Joint multi-resource day-ahead probabilistic forecast model section by lower Ignorance Score (IS). (a) Energy Score (ES), (b) Variogram Score with $p = 0.5$ ($VS^{0.5}$), and (c) IS. (d) Skill Score based on RMSE (SS_{RMSE}) relative to CAISO's forecast, and (e) training and testing computing time.

Supplementary Tables

Supplementary Table 1: This table contains the results of Forecast Skill Scores (SS) for independent (Fig. 2b) and joint (Fig. 2e) day-ahead forecasts for each energy feature aggregated across nodes (SS ↑ is better).

	**Kernel**	**Load**	**Solar**	**Wind**
	**Lasso**			
BLR		2.36	12.13	-1.48
RVM		-8.8	-4.67	-1.55
GPR	$\mathcal{K}_L$, $\mathcal{K}_L$, $\mathcal{K}_{RQ}$	1.17	10.99	3
SLGPR	$\mathcal{K}_L$, $\mathcal{K}_L$, $\mathcal{K}_{M_{1.5}}$	-7.68	11.8	4.67
NLGPR	$\mathcal{K}_L$, $\mathcal{K}_L$, $\mathcal{K}_L$	**6.13**	15.79	0.75
	**OMP**			
BLR		-0.01	9.48	-1.71
RVM		-13.38	-0.68	-1.18
GPR	$\mathcal{K}_L$, $\mathcal{K}_L$, $\mathcal{K}_{RQ}$	-0.04	12.49	1.58
SLGPR	$\mathcal{K}_L$, $\mathcal{K}_L$, $\mathcal{K}_{M_{2.5}}$	2.73	9.8	1.47
NLGPR	$\mathcal{K}_L$, $\mathcal{K}_L$, $\mathcal{K}_L$	4.55	13.07	1.17
	**Elastic Net**			
BLR		4.15	12.17	-1.77
RVM		-9.95	1.09	0.58
GPR	$\mathcal{K}_{RQ}$, $\mathcal{K}_L$, $\mathcal{K}_{RQ}$	-17.97	11.66	4.95
SLGPR	$\mathcal{K}_L$, $\mathcal{K}_L$, $\mathcal{K}_{M_{1.5}}$	-9.56	11.86	**5.94**
NLGPR	$\mathcal{K}_L$	5.84	**16.77**	1.9
	**Group Lasso**			
BLR		-14.02	13.73	-1.44
RVM		-16.5	10.54	-3.75
GPR	$\mathcal{K}_{RQ}$, $\mathcal{K}_L$, $\mathcal{K}_{RQ}$	-22.3	12.79	4.53
SLGPR	$\mathcal{K}_L$, $\mathcal{K}_L$, $\mathcal{K}_{M_{1.5}}$	-15.36	12.05	5.14
NLGPR	$\mathcal{K}_L$	-7.1	13.43	-7.21

Supplementary Table 2: This table contains the results of net demand independent (Fig. 2e) and joint (Fig. 2f) probabilistic forecast SS at nodal and system levels (SS ↑ is better).

	**Kernel**	**CAISO**	**NP15**	**SP15**	**ZP26**
		**Lasso**			
BLR		22.92	19.25	13.88	7.85
RVM		9.96	1.69	-1.99	-16.78
GPR	$\mathcal{K}_L$,$\mathcal{K}_L$,$\mathcal{K}_{RQ}$	21.8	19.18	12.32	6.98
SLGPR	$\mathcal{K}_L$,$\mathcal{K}_L$,$\mathcal{K}_{M_{1.5}}$	21.04	13.38	14.76	9.58
NLGPR	$\mathcal{K}_L$	24.81	16.6	13.79	8.65
		**OMP**			
BLR		23.5	11.58	**16.67**	9.15
RVM		13.92	-0.1	0.79	-7.05
GPR	$\mathcal{K}_L$,$\mathcal{K}_L$,$\mathcal{K}_{RQ}$	24.25	11.69	16.57	8.96
SLGPR	$\mathcal{K}_L$,$\mathcal{K}_L$,$\mathcal{K}_{M_{2.5}}$	23.43	14.23	16.3	9.94
NLGPR	$\mathcal{K}_L$	23.11	12.44	13.61	**10.14**
		**Elastic Net**			
BLR		**25.23**	**19.4**	16.38	9.02
RVM		11.23	0.66	0.7	-12.01
GPR	$\mathcal{K}_{RQ}$,$\mathcal{K}_L$,$\mathcal{K}_{RQ}$	9.73	4.03	6.26	-0.25
SLGPR	$\mathcal{K}_L$,$\mathcal{K}_L$,$\mathcal{K}_{M_{1.5}}$	20.7	12.43	15.34	9.22
NLGPR	$\mathcal{K}_L$	24.39	16.3	12.86	9.26
		**Group Lasso**			
BLR		18.62	5.9	12.65	9.44
RVM		12.13	-13.66	3.15	-8.21
GPR	$\mathcal{K}_{RQ}$,$\mathcal{K}_L$,$\mathcal{K}_{RQ}$	9.16	1.21	6.05	-5.39
SLGPR	$\mathcal{K}_L$,$\mathcal{K}_L$,$\mathcal{K}_{M_{1.5}}$	19	7.75	13.46	9.04
NLGPR	$\mathcal{K}_L$	12.99	0.83	2.82	7.19

Supplementary Table 3: Scores achieved by the different day-ahead probability forecasting models of electricity demand (Fig. 5a, d, and g), solar generation (Fig. 5b, e, and h), and wind generation (Fig. 5c, f, and i). The scores are Energy Score (ES), Variogram Score ($VS^{0.5}$), Interval Score (IS), Skill Score (SS_{RMSE}) compared to CAISO's forecast, and computational time.

**Sparse Learning**	**Dense Learning**	**Kernel**	**Feature**	**RMSE**	**FS**	**ES**	**VS**	**IS**	**Tr. Time**	**Ts. Time**
Lasso	BLR		load	1104.12	2.36	39.75	2380.29	8163.84	116.70	0.80
Lasso	BLR		solar	616.20	12.13	33.63	2214.89	5454.18	1070.64	2.94
Lasso	BLR		wind	449.21	-1.48	31.78	2330.46	4809.18	113.95	0.85
Lasso	GPR	$\mathcal{K}_L$	load	1117.54	1.17	40.40	2465.06	8324.47	834.35	0.99
Lasso	GPR	$\mathcal{K}_L$	solar	624.19	10.99	33.00	2279.55	5452.95	3538.41	2.48
Lasso	GPR	$\mathcal{K}_{RQ}$	wind	429.37	3.00	31.53	2186.33	4980.05	3289.84	0.92
Lasso	RVM		load	1230.27	-8.80	48.83	3633.57	20340.11	1122.05	1.30
Lasso	RVM		solar	734.03	-4.67	37.52	2943.02	7762.18	644.41	0.84
Lasso	RVM		wind	449.50	-1.55	32.24	2731.55	4967.20	29.49	0.81
Lasso	SLGPR	$\mathcal{K}_{M_{1.5}}$	wind	422.00	4.67	31.37	2182.85	5668.83	2942.37	5.45
Lasso	SLGPR	$\mathcal{K}_L$	load	1217.63	-7.68	42.64	2505.08	11011.55	3346.59	11.29
Lasso	SLGPR	$\mathcal{K}_L$	solar	618.52	11.80	33.15	2117.53	6760.84	6511.18	10.05
OMP	BLR		load	1130.85	-0.01	41.93	2654.42	8428.81	19.47	0.89
OMP	BLR		solar	634.76	9.48	33.65	2329.27	5415.37	45.59	1.17
OMP	BLR		wind	450.21	-1.71	31.82	2334.30	4805.96	15.02	1.00
OMP	GPR	$\mathcal{K}_L$	load	1131.19	-0.04	42.31	2708.22	8385.01	503.95	0.93
OMP	GPR	$\mathcal{K}_L$	solar	613.68	12.49	32.65	2351.23	4628.44	896.46	0.95
OMP	GPR	$\mathcal{K}_{RQ}$	wind	435.65	1.58	32.15	2229.28	4930.26	4033.00	1.13
OMP	RVM		load	1282.02	-13.38	62.00	5704.26	14714.92	112.41	0.81
OMP	RVM		solar	706.04	-0.68	37.23	2910.84	6972.10	23.14	0.82
OMP	RVM		wind	447.87	-1.18	32.43	2791.95	4883.68	2.59	0.80
OMP	SLGPR	$\mathcal{K}_{M_{2.5}}$	wind	436.16	1.47	32.56	2253.62	5720.02	2468.53	4.82
OMP	SLGPR	$\mathcal{K}_L$	load	1099.94	2.73	40.45	2483.98	9424.48	3251.94	11.08
OMP	SLGPR	$\mathcal{K}_L$	solar	632.53	9.80	33.50	2119.39	7060.30	6817.35	17.01
Elastic Net	BLR		load	1083.79	4.15	39.32	2394.04	8087.04	80.52	0.81
Elastic Net	BLR		solar	615.89	12.17	33.97	2197.84	5444.52	913.85	5.84
Elastic Net	BLR		wind	450.48	-1.77	31.91	2336.52	4820.43	148.19	1.08
Elastic Net	GPR	$\mathcal{K}_{RQ}$	load	1333.92	-17.97	43.05	2534.89	18705.54	6420.78	1.26
Elastic Net	GPR	$\mathcal{K}_L$	solar	619.51	11.66	33.03	2262.97	5197.78	4590.76	3.12
Elastic Net	GPR	$\mathcal{K}_{RQ}$	wind	420.76	4.95	30.83	2135.10	4942.62	2860.59	0.90
Elastic Net	RVM		load	1243.30	-9.95	49.50	3697.87	20951.83	1252.71	1.47
Elastic Net	RVM		solar	693.65	1.09	35.87	2747.27	7061.39	1401.97	1.18
Elastic Net	RVM		wind	440.09	0.58	31.87	2679.50	4899.63	28.87	0.72
Elastic Net	SLGPR	$\mathcal{K}_L$	load	1238.88	-9.56	42.99	2540.53	11531.38	3520.53	11.55
Elastic Net	SLGPR	$\mathcal{K}_L$	solar	618.11	11.86	33.98	2177.11	6447.40	11310.36	11.19
Elastic Net	SLGPR	$\mathcal{K}_{M_{1.5}}$	wind	416.34	5.94	30.75	2144.09	5615.62	2622.51	4.58
Group Lasso	BLR		load	1289.34	-14.02	47.43	3088.95	9637.66	3943.20	0.76
Group Lasso	BLR		solar	604.97	13.73	32.49	2211.55	4829.10	7353.41	1.43
Group Lasso	BLR		wind	449.04	-1.44	31.87	2358.61	4847.03	558.66	0.93
Group Lasso	GPR	$\mathcal{K}_{RQ}$	load	1382.91	-22.30	44.16	2680.14	19057.39	9022.40	0.99
Group Lasso	GPR	$\mathcal{K}_L$	solar	611.54	12.79	32.18	2275.40	5153.34	6159.04	1.25
Group Lasso	GPR	$\mathcal{K}_{RQ}$	wind	422.61	4.53	30.99	2135.04	4716.95	3831.63	0.80
Group Lasso	RVM		load	1317.32	-16.50	56.15	4580.24	20546.85	5210.57	0.94
Group Lasso	RVM		solar	627.34	10.54	34.97	2776.67	4888.73	5058.80	0.74
Group Lasso	RVM		wind	459.27	-3.75	32.94	2836.01	4979.35	581.40	0.72
Group Lasso	SLGPR	$\mathcal{K}_L$	load	1304.44	-15.36	47.26	2950.27	11824.63	6883.39	9.83
Group Lasso	SLGPR	$\mathcal{K}_L$	solar	616.73	12.05	33.53	2153.48	6618.87	11917.14	9.47
Group Lasso	SLGPR	$\mathcal{K}_{M_{1.5}}$	wind	419.90	5.14	31.06	2158.79	5664.73	4482.32	8.28

Supplementary Table 4: Scores achieved by the proposed forecasting models when evaluated at the system level (Fig. 5a-e): Energy Score (ES), Variogram Score ($VS^{0.5}$), Interval Score (IS), Skill Score (SS_{RMSE}) compared to CAISO's forecast, and computational time.

**Sparse Learning**	**Dense Learning**	**Kernel**	**RMSE**	**FS**	**ES**	**VS**	**IS**	**Tr. Time**	**Ts. Time**
Lasso	BLR		1349.56	22.92	86.61	21845.28	14740.34	1301.29	4.59
Lasso	RVM		1576.58	9.96	100.97	28707.23	26310.88	1795.95	2.95
Lasso	GPR	$\mathcal{K}_L$,$\mathcal{K}_L$,$\mathcal{K}_{RQ}$	1369.25	21.8	86.78	21871.29	15184.85	7662.6	4.39
Lasso	SLGPR	$\mathcal{K}_L$,$\mathcal{K}_L$,$\mathcal{K}_{M_{1.5}}$	1382.44	21.04	89.61	21528.81	17575.56	12800.15	26.79
Lasso	NLGPR	$\mathcal{K}_L$	1316.5	24.81	85.34	21800.45	14016.98	10817.48	24.47
OMP	BLR		1339.45	23.5	88.98	22692.35	14413.46	80.08	3.06
OMP	RVM		1507.14	13.92	115.77	32591.66	20659.4	138.14	2.42
OMP	GPR	$\mathcal{K}_L$,$\mathcal{K}_L$,$\mathcal{K}_{RQ}$	1326.29	24.25	88.32	22467.54	14304.54	5433.4	3
OMP	SLGPR	$\mathcal{K}_L$,$\mathcal{K}_L$,$\mathcal{K}_{M_{1.5}}$	1340.6	23.43	87.62	21694.01	16650.82	12537.82	32.9
OMP	NLGPR	$\mathcal{K}_L$	1346.3	23.11	87.02	22429.9	14488.85	9557.45	24.51
Elastic Net	BLR		1309.16	25.23	85.94	21667.17	14009.14	1142.57	7.72
Elastic Net	RVM		1554.29	11.23	100	27840.94	25955.55	2683.55	3.38
Elastic Net	GPR	$\mathcal{K}_{RQ}$,$\mathcal{K}_L$,$\mathcal{K}_{RQ}$	1580.55	9.73	91.71	22264.07	26499.03	13872.13	5.27
Elastic Net	SLGPR	$\mathcal{K}_L$,$\mathcal{K}_L$,$\mathcal{K}_{M_{1.5}}$	1388.48	20.7	90.37	21558.69	17739.86	17453.4	27.33
Elastic Net	NLGPR	$\mathcal{K}_L$,$\mathcal{K}_L$,$\mathcal{K}_L$	1323.91	24.39	85.46	21815.17	14128.5	10989.06	24.15
Group Lasso	BLR		1424.91	18.62	94.18	23506.19	15456.15	11855.26	3.12
Group Lasso	RVM		1538.54	12.13	107.33	29836.69	22977.3	10850.77	2.39
Group Lasso	GPR	$\mathcal{K}_{RQ}$,$\mathcal{K}_L$,$\mathcal{K}_{RQ}$	1590.48	9.16	92.23	22659.52	25706.81	19013.06	3.05
Group Lasso	SLGPR	$\mathcal{K}_L$,$\mathcal{K}_L$,$\mathcal{K}_{M_{1.5}}$	1418.19	19	95.12	22593.11	17671.88	23282.86	27.57
Group Lasso	NLGPR	$\mathcal{K}_L$	1523.46	12.99	92.54	24772	16861.12	79001.78	29.02

Supplementary Notes

Supplementary Note 1

The methods proposed in this investigation are fully probabilistic and provide a predictive Probability Density Function (PDF). In addition, the proposed methods can provide ensemble forecasts in a complementary manner. However, it is necessary to apply an appropriate scoring rule to evaluate each approach properly [5, 6]. A scoring rule is a function of a predictive PDF $\hat{f}$, and a realized observation y [7]. The objective of the scoring rule is to perform a *quantitative* evaluation of a forecasting method. The suitable scoring rules are selected from a compilation of proper scoring rules developed for day-ahead resource forecasting [8].

Skill Score (SS). This score measures the improvement of a forecast over a baseline or reference forecast. The SS is a commonly used scoring rule in weather forecasting that is based on another scoring rule. We use the SS to calibrate the location of the predictive mean $\hat{\boldsymbol{\mu}}_{k,t}$ with respect to the baseline point-wise forecast. Root Mean Squared Error (RMSE) is preferable in our task since it weighs large errors heavier than other deterministic metrics (e.g., mean absolute error). The SS is,

$$\begin{aligned} \mathrm{SS}_{\mathbf{y}}\left(\hat{\mathbf{y}}_{k,t}, \mathbf{y}_{k,t}\right) &= 100 \cdot \left(1 - \frac{\mathrm{RMSE}_{\mathbf{y}}\left(\hat{\mathbf{y}}_{k,t}, \mathbf{y}_{k,t}\right)}{\mathrm{RMSE}_{\mathrm{baseline}}}\right), \\ \mathrm{RMSE}_{\mathbf{y}}\left(\hat{\mathbf{y}}_{k,t}, \mathbf{y}_{k,t}\right) &= \sum_{t=1}^{T} \sqrt{\frac{1}{K} \left\| \hat{\mathbf{y}}_t - \mathbf{y}_t \right\|^2}. \end{aligned} \tag{1}$$

where $\| \cdot \|$ is the ℓ_2-norm, $\hat{y}_{k,t}$ is a point-wise forecast and $y_{k,t}$ is the actual observation ($\hat{\mathbf{y}}_{k,t} = \hat{\boldsymbol{\mu}}_{k,t}$ in a probabilistic forecast); the index h represent the hour in day d and spatial region t. A positive SS means a performance improvement, while a negative one is a reduction. A perfect forecast is 100, and no improvement with respect to the baseline is 0.

Energy Score (ES). This scoring rule is the multivariate generalization of the Continuous Rank Probability Score [9]. IS is accessible in forecasting methods that provide a predictive density. In this context, ES is ideal for ensemble forecasts generated by an approach based on the Monte Carlo Markov chain. ES is defined as,

$$\begin{aligned} \mathrm{ES}_{\mathbf{y}}\left(\hat{F}_{k,t}, \mathbf{y}_{k,t}\right) &= \int_{-\infty}^{\infty} \left(\hat{F}_{k,t}\left(\mathbf{u}\right) - \mathbb{I}\left(\mathbf{u} \geq \mathbf{y}_{k,t}\right)\right)^2 \mathrm{d}\mathbf{u} \\ &= \frac{1}{M} \sum_{j=1}^{M} \left\| \hat{\mathbf{y}}_{k,t,j} - \mathbf{y}_{k,t} \right\| - \frac{1}{2M^2} \sum_{j=1}^{M} \sum_{k=1}^{M} \left\| \hat{\mathbf{y}}_{k,t,j} - \hat{\mathbf{y}}_{k,t,k} \right\|, \end{aligned} \tag{2}$$

where $\hat{F}_{k,t}$ is the predictive CDF for hour h in day d; $\| \cdot \|$ is the ℓ_2-norm, and M is the number of forecasts in the ensamble. It is important to mention that the ensembles are drawn for the predictive probability that depends on $\hat{\mathbf{y}}_{h-1,d}$, the ensemble drawn for the previous hour,

$$\hat{\mathbf{y}}_{k,t,j} \sim p\left(\hat{\mathbf{y}}_{k,t} \middle| \hat{\boldsymbol{\mu}}_{k,t}, \hat{\boldsymbol{\Sigma}}_{k,t}, \hat{\mathbf{y}}_{h-1,d,j}, \ldots, \hat{\mathbf{y}}_{1,d,j}\right). \tag{3}$$

ES measures the squared distance between $\hat{\mathbf{y}}_{k,t,j}$ an ensemble forecast and $\mathbf{y}_{k,t}$, the observed performance, and is considered numerically more robust than LogS because it is based on CDF instead of PDF. The lower $\mathrm{ES}_{\mathbf{y}}$ is the better the model represents the correlation between the horizons of the ensemble scenarios.

Variogram Score ($\mathbf{VS}^p$). Another scoring rule suitable for multivariate ensemble forecasts is the VS of order p [7]. This scoring rule, also named the structure function [10], correctly detects the correlation structure in multivariate

predictions. It is defined as,

$$\text{VS}_{\mathbf{y}}^{p}\left(\hat{F}_{k,t}, \mathbf{y}_{k,t}\right) = \sum_{t=1}^{T}\sum_{t'=1}^{T}\left(|y_{h,d,t} - y_{h,d,t'}|^{p} - \frac{1}{M}\sum_{j=1}^{M}|\hat{y}_{h,d,t,j} - \hat{y}_{h,d,t',j}|^{p}\right)^{2} \tag{4}$$

where $|\cdot|$ denotes the absolute value. The parameter p transforms the distribution of absolute differences and improves separability. We use $p = 0.5$ as it was found to be the most adequate choice for Gaussian distributions [10]. The lower $\text{VS}_{\mathbf{y}}^{0.5}$ is the better.

Interval Score (IS). This score is important for quantile prediction. However, we can use it to evaluate the accuracy of different predictive intervals in a fully probabilistic forecast. A lower IS indicates that a model approximates more accurately a predictive interval $1 - \alpha$,

$$\begin{aligned}\text{IS}_{1-\alpha}\left(\hat{f}_{k,t}, \mathbf{y}_{k,t}, z, \alpha\right) = 2z\hat{\boldsymbol{\sigma}}_{k,t} &+ \frac{2}{\alpha}\left(\hat{\boldsymbol{\mu}}_{k,t} - z\hat{\boldsymbol{\sigma}}_{k,t} - \mathbf{y}_{k,t}\right)\mathbb{I}\left(\mathbf{y}_{k,t} < \hat{\boldsymbol{\mu}}_{k,t} - z\hat{\boldsymbol{\sigma}}_{k,t}\right) \\ &+ \frac{2}{\alpha}\left(\mathbf{y}_{k,t} - \hat{\boldsymbol{\mu}}_{k,t} + z\hat{\boldsymbol{\sigma}}_{k,t}\right)\mathbb{I}\left(\mathbf{y}_{k,t} > \hat{\boldsymbol{\mu}}_{k,t} + z\hat{\boldsymbol{\sigma}}_{k,t}\right),\end{aligned} \tag{5}$$

where the indicator function $\mathbb{I}(\cdot)$ is counts the number of samples outside the prediction interval; $\hat{\boldsymbol{\mu}}_{k,t}$ and $\hat{\boldsymbol{\sigma}}_{k,t}$ are the predictive mean and standard divination; α is the p-value and z is the z-score of a given prediction interval. For example, a prediction interval 95% has $\alpha = 0.05$ and $z = 1.959$. The lower $\text{IS}_{1-\alpha}$ is the better. The forecast is rewarded in narrow intervals but penalized when an observation is outside.

In addition to the above scores, there are quartile-based scoring rules and diagnosis histograms [8]. However, the proposed methods are not based on quantile regression. The forecast is obtained from a fully probabilistic model and provides a predictive density. For that reason, we consider the quantile and interval scoring rules to be inappropriate in this investigation.

Supplementary Note 2

The kernel functions in this analysis are linear (L), polynomial of order n (P^n), Radial Basis Function (RBF), Rational Quadratic (RQ), and Matérn (M_ν). Their respective functions are,

$$\begin{aligned}\mathcal{K}_{L}\left(\mathbf{x}_i, \mathbf{x}_j\right) &= \theta_1 \mathbf{x}_i^{\top}\mathbf{x}_j + \theta_2, \\ \mathcal{K}_{P^n}\left(\mathbf{x}_i, \mathbf{x}_j\right) &= \left(\theta_3 \mathbf{x}_i^{\top}\mathbf{x}_j + \theta_4\right)^{n}, \\ \mathcal{K}_{RBF}\left(\mathbf{x}_i, \mathbf{x}_j\right) &= \exp\left(-\theta_5||\mathbf{x}_i - \mathbf{x}_j||^2\right) + \theta_6, \\ \mathcal{K}_{RQ}\left(\mathbf{x}_i, \mathbf{x}_j\right) &= \left(1 + \frac{1}{2\theta_7\theta_8^2}||\mathbf{x}_i - \mathbf{x}_j||^2\right)^{-\theta_7} + \theta_9, \\ \mathcal{K}_{M_\nu}\left(\mathbf{x}_i, \mathbf{x}_j\right) &= \frac{2^{1-\nu}}{\Gamma(\nu)}\left(\sqrt{2\nu}\cdot\theta_{10}||\mathbf{x}_i - \mathbf{x}_j||^2\right)^{\nu} K_{\nu}\left(\sqrt{2\nu}\cdot\theta_{10}||\mathbf{x}_i - \mathbf{x}_j||^2\right) + \theta_{11},\end{aligned} \tag{6}$$

where $\{\theta_1, \ldots, \theta_{11}\} \in \mathbb{R}^{+}$, the Matérn order $\nu = \{0.5, 1.5, 2.5\}$, and the polynomial degrees $n = \{2, 3\}$ are hyperparameters [11]. $\Gamma(\cdot)$ is the Gamma function, and K_ν is the modified Bessel function of second kind (see further information about the kernel parameters in Table 5).

Supplementary Note 3

NOAA Operational Model Archive and Distribution System (NOMADS). RAP and HRRR are operational models. The Rapid Refresh is the continental-scale NOAA hourly-updated assimilation/modeling system operational. RAP covers North America and is comprised primarily of a numerical weather forecast and an analysis/assimilation

Supplementary Table 5: Summary of the different parameters in each kernel function and their respective roles.

**Kernel**	**Function**	**Type**	**Parameters**	**Description**
Linear (L)	$\mathcal{K}_L\left(\mathbf{x}_i, \mathbf{x}_j\right)$	Non-stationary	scale (θ_1) and bias (θ_2)	Low complexity linear transformation with scale (θ_1) and bias parameters (θ_2)—alternative formulation to no-scale and no-bias
Polynomial (P^n)	$\mathcal{K}_{P^n}\left(\mathbf{x}_i, \mathbf{x}_j\right)$	Non-stationary	Amplitude (θ_3), bias (θ_4), and polynomial order (n)	An efficient polynomial expansion formulation that avoid its explicit computation in a Hilbert space
Radial Basis Function (RBF)	$\mathcal{K}_{RBF}\left(\mathbf{x}_i, \mathbf{x}_j\right)$	Stationary	Length-scale (θ_5) and bias (θ_6)	Non-linear kernel based on similarity metric between samples tuned by the length-scale parameter (as $\theta_5 \to \infty$ the RBF concentrates the weight on a single sample)
Rational Quadratic (RQ)	$\mathcal{K}_{RQ}\left(\mathbf{x}_i, \mathbf{x}_j\right)$	Stationary	Scale (θ_7), length-scale (θ_8), and bias (θ_9)	Scaled mixtures of RBF kernels (as $\theta_7 \to \infty$ the RQ converges to a RBF kernel)
Matérn (M_ν)	$\mathcal{K}_{M_\nu}\left(\mathbf{x}_i, \mathbf{x}_j\right)$	Stationary	Length-scale (θ_{10}), bias (θ_{11}), and Gamma function order (ν)	Improves smoothness control over RBF ($\nu = 0.5$ is an exponential kernel—less smooth—, and as $\nu \to \infty$ converges to a RBF kernel—more smooth)

system to initialize that model. RAPv5 implemented from Wed. Dec 2, 2020, at NCEP. RAP model website is `https://rapidrefresh.noaa.gov`.

RAP is complemented by the higher-resolution High-Resolution Rapid Refresh (HRRR) model, which is updated hourly and covers a smaller geographic domain. The HRRR is a NOAA real-time 3 km resolution, hourly updated, cloud-resolving, convection-allowing atmospheric model, initialized by 3 km grids with 3 km radar assimilation. Radar data is assimilated in the HRRR every 15 min over 1 hour adding further detail to that provided by the hourly data assimilation from the 13 km radar-enhanced Rapid Refresh. HRRR model website is `https://rapidrefresh.noaa.gov/hrrr/`.

HRRRv4 provides a 48-hour forecast (i.e. cycle) every 6 hours (00, 06, 12, and 18 UTC) from 2 Dec, 2020. HRRRv3 (available from 12 Jul, 2018) does not have a 48-hour forecast every 6 hours, only a 6-hour forecast every hour. Python API instructions are in `https://mesowest.utah.edu/html/hrrr/`.

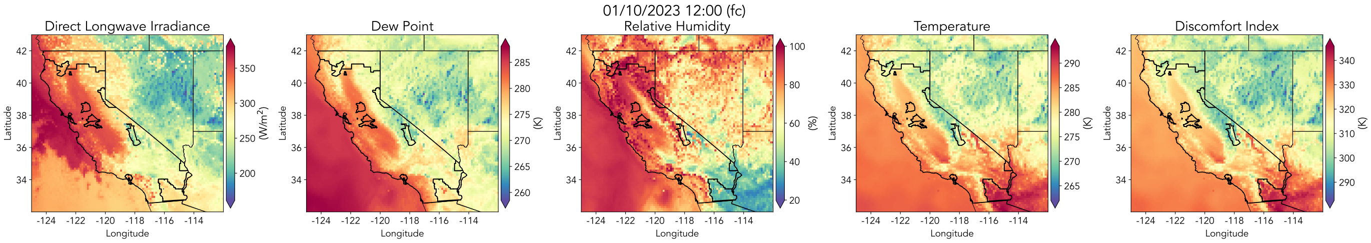

Supplementary Figure 20: Weather features in the electric load forecast from the HRRR forecast at 4 pm PTZ for noon the next day. Panels from left to right direct long-ware irradiance (surface), dew point (surface), relative humidity (surface), and discomfort index (2 m). Panels from left to right in the bottom row, relative humidity (2 m), temperature (2 m), and wind speed at 10 m and 80 m. Map data © Esri; sources: California Energy Commission and Natural Earth [3, 4].

Wind Velocity Extrapolation at Different Heights. HRRR operation forecast only includes wind components estimated at 10 m and 80 m above sea level. However, research in the literature reports 60 m, 80 m, 100 m, and 120 m as effective heights for a wind operational forecast.

The wind profile can be estimated by applying the Power Law [12]. The power low requires knowing the wind velocity components $\mathrm{U}_{i,j}$ and $\mathrm{V}_{i,j}$ at two different heights. We use the velocity magnitude at 10 m ($\mathrm{W}_{10,i,j}$) and 80 m

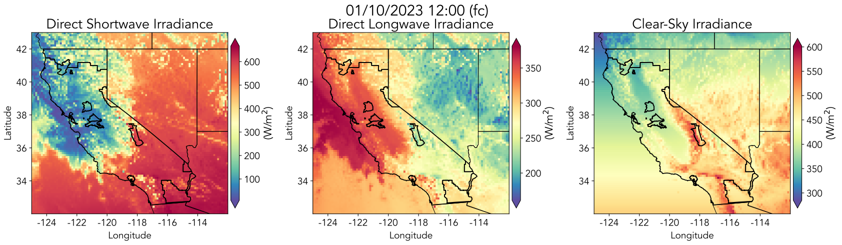

Supplementary Figure 21: Weather features in the solar generation forecast from the HRRR forecast at 4 pm PTZ for noon the next day. Panels from left to right direct short-wave irradiance (surface), direct long-wave irradiance (surface), and clear-sky irradiance (surface). Map data © Esri; sources: California Energy Commission and Natural Earth [3, 4].

($\mathrm{W}_{80,i,j}$), to calculate the α parameter in the power law,

$$\alpha_{i,j} = \frac{\log \mathrm{W}_{80,i,j} - \log \mathrm{W}_{10,i,j}}{\log 80 - \log 10}. \tag{7}$$

The wind velocity magnitude at 60 m ($\mathrm{W}_{60,i,j}$), 100 m ($\mathrm{W}_{100,i,j}$) and 120 m ($\mathrm{W}_{120,i,j}$) for each i, j point in the $M \times N$ grid are

$$\begin{aligned} \mathrm{W}_{60,i,j} &= \mathrm{W}_{10,i,j} \left(\frac{10}{60}\right)^{\alpha_{i,j}}, \\ \mathrm{W}_{100,i,j} &= \mathrm{W}_{80,i,j} \left(\frac{80}{100}\right)^{\alpha_{i,j}}, \\ \mathrm{W}_{120,i,j} &= \mathrm{W}_{80,i,j} \left(\frac{80}{120}\right)^{\alpha_{i,j}}. \end{aligned} \tag{8}$$

See the results in the first three panels in Supplementary Figure 22.

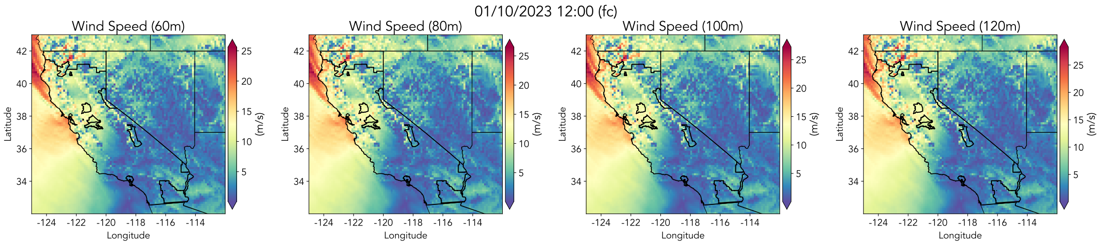

Supplementary Figure 22: Panels from left to right, derive wind speed at 60 m, 100 m, 120 m, and clear-sky direct irradiance. The wind speeds were derived wind speed at 10 m and 80 m obtained from HRRR weather forecast at 4 pm PTZ for noon the next day. These weather features are used in the wind generation forecast. Map data © Esri; sources: California Energy Commission and Natural Earth [3, 4].

Apparent Temperature Features. In an operational forecast of load, some metrics have been reported to increase the performances: Discomfort Index (DI) [13] and Wind Chill (WC), HDD, and CDD. The formulas to compute each one of these metrics are here.

Discomfort Index:

$$\mathrm{DI}_{i,j} = (1.8\mathrm{T}_{i,j} + 32) + [(0.55 - 0.0055\mathrm{RH}_{i,j})(1.8\mathrm{T}_{i,j} - 26)] \tag{9}$$

where $\mathrm{T}_{i,j}$ (K) is the air temperature, and $\mathrm{RH}_{i,j}$ (%) is the relative humidity. $\mathrm{DI}_{i,j}$ (°C) is a temperature measure.

Wind Chill:

$$\mathrm{WC}_{i,j} = 13.12 + 0.06215\mathrm{T}_{i,j} - 11.37\mathrm{W}_{10,i,j}^{0.16} + 0.3965\mathrm{T}_{i,j}\mathrm{W}_{10,i,j}^{0.16} \tag{10}$$

where $\mathrm{W}_{10,i,j}$ (km/h) is the wind velocity magnitude at 10 m, $\mathrm{T}_{i,j}$ (K) is the air temperature, and $\mathrm{WC}_{i,j}$ (K) is a

temperature measure.

Heating Degree Day (HDD):

$$\mathrm{HDD}_{i,j} = \begin{cases} \mathrm{T}_{i,j} - 65 & \mathrm{T}_{i,j} - 65 < 0 \\ 0 & \text{Otherwise,} \end{cases} \tag{11}$$

where $\mathrm{T}_{i,j}$ (°F) is the air temperature. Cooling Degree Day (CDD):

$$\mathrm{CDD}_{i,j} = \begin{cases} \mathrm{T}_{i,j} - 65 & \mathrm{T}_{i,j} - 65 > 0 \\ 0 & \text{Otherwise,} \end{cases} \tag{12}$$

where $\mathrm{T}_{i,j}$ (°F) is the air temperature. Opposite to HDD.

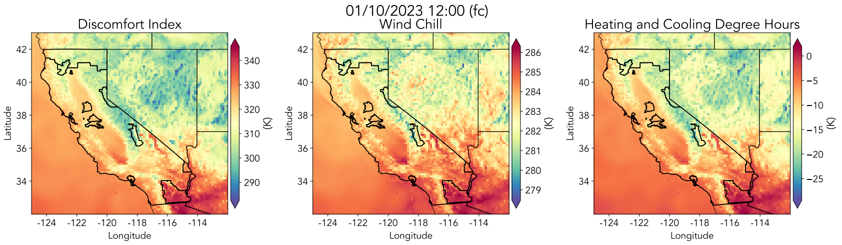

Supplementary Figure 23: From left to right, the panels show the discomfort index, wind chill, and heating and cooling degree hours derived from weather features obtained from the HRRR weather forecast at 4 pm PTZ for noon the next day. Map data © Esri; sources: California Energy Commission and Natural Earth [3, 4].

Supplementary Note 4

The spatial counts of energy features in a given neighborhood define the points (i.e., pixel) in a grid with weather features, a priori, more informative. For that, consider a point in a grid $\mathbf{x}'_{i,j}$ defined by a pair of spatial coordinates, latitude $\phi_{i,j}$ and longitude $\lambda_{i,j}$. Similarly, the k-th location $\mathbf{x}'_k$ of energy feature $\mathcal{X}$ has latitude ϕ_k and longitude λ_k coordinates.

$$m^{\mathcal{E}}_{i,j} \triangleq \begin{cases} m^{\mathcal{X}}_{i,j} + 1 & \|\mathbf{x}'_{i,j} - \mathbf{x}^{\mathcal{X}}_k\|_2 \geq \sigma \\ m^{\mathcal{X}}_{i,j} & \text{Otherwise.} \end{cases} \qquad \forall k = 1, \dots, D^{\mathcal{X}}. \tag{13}$$

The parameter $\sigma = 0.25$ controls the neighborhood pixel size used in the counting. The result is spatial density filter $\mathbf{M}^{\mathcal{X}} \in \mathbb{N}^{M \times N}$ for each energy feature $\mathcal{X}$, see in Supplementary Figure 24.

Supplementary Note 5

The weather features obtained from the NOAA operational forecast are: atmospheric pressure ($\mathrm{AP}_{i,j}$), direct short-wave irradiance ($\mathrm{I}_{i,j}$), direct long-wave irradiance ($\mathrm{R}_{i,j}$), dew point ($\mathrm{D}_{i,j}$), relative humidity ($\mathrm{H}_{i,j}$), air temperature ($\mathrm{T}_{i,j}$), wind velocity components at 10 m ($\mathrm{U}_{10,i,j}$ and $\mathrm{V}_{10,i,j}$) and 80 m ($\mathrm{U}_{80,i,j}$ and $\mathrm{V}_{80,i,j}$).

Other weather features are derived from the NOAA operational forecast. The wind velocity components are transformed to wind velocity magnitudes $\mathrm{W}^{10}_{i,j}$ and $\mathrm{W}^{80}_{i,j}$, and used to derived $\mathrm{W}^{60}_{i,j}$, $\mathrm{W}^{100}_{i,j}$ and $\mathrm{W}^{120}_{i,j}$ applying the power law. The coordinates $\mathbf{x}_{i,j}$ and the elevation $\eta_{i,j}$ (from GMTED2010 dataset) of each point on the grid are used to derive the clear-sky irradiance ($G_{i,j}$). The air temperature ($\mathrm{T}_{i,j}$) and the relative humidity ($\mathrm{H}_{i,j}$) is used to derived the Discomfort Index ($\mathrm{DI}_{i,j}$). Similarly, the air temperature ($\mathrm{T}_{i,j}$) and the wind velocity magnitude ($\mathrm{W}^{10}_{i,j}$) is used to derived the Wind Chill ($\mathrm{WC}_{i,j}$).

A weather feature $X_{i,j,d}$ is defined by two spatial indexes i, j (grid coordinates) and a time index d (operational day). An observed weather feature can be an actual $X_{i,j,d}$ or a prediction $\hat{X}_{i,j,k,t}$ in a 24-hour forecast. The time horizon in the 24-hour forecast is $h = \ell + \rho$, where the day hour is $\rho = \{1, \dots, 24\}$ and the lead time is $\ell = 8$. After

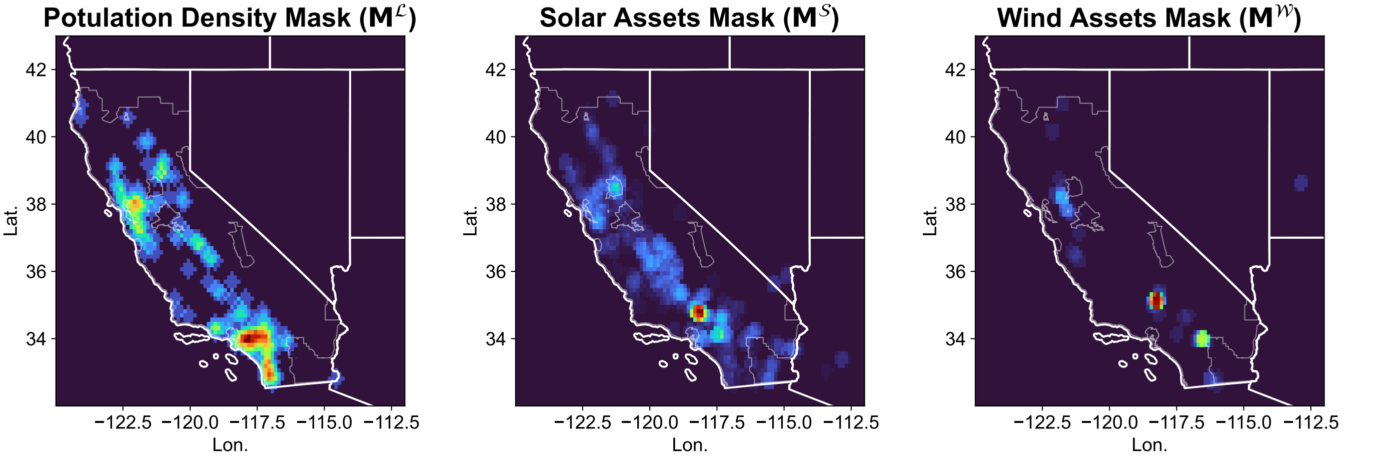

Supplementary Figure 24: From left to right, spatial density for electricity demand (i.e. population density) $\mathcal{L}$, solar energy supply $\mathcal{S}$, and wind energy supply $\mathcal{W}$. The fewer counts, the darker the color. The more the counts, the redder the color becomes. Map data © Esri; sources: California Energy Commission and Natural Earth [3, 4].

applying the spatial filtering to a weather feature's actual $X_{i,j,d}$ or prediction $\hat{X}_{i,j,k,t}$, the structure has vector form $\mathbf{x}^{\mathcal{X}}_{k,t} = \{\mathrm{X}_{i,j,k,t} \mid m^{\mathcal{X}}_{i,j} > 0\}$ with dimensions $\mathbf{x}^{\mathcal{X}}_{k,t} \in \mathbb{R}^{D^{\mathcal{X}}}$, where $D^{\mathcal{X}} = \sum_{i,j} \mathbb{I}\left(m^{\mathcal{X}}_{i,j} > 0\right)$ and $\mathbb{I}(\cdot)$ is the indicator function. It applies to $\hat{\mathbf{x}}^{\mathcal{X}}_{k,t}$ in the same manner. The resulting weather feature vectors for each day d are summarized and grouped into different categories:

Irradiance features: clear-sky ($\mathbf{g}^{\mathcal{X}}_{k,t}$ and $\hat{\mathbf{g}}^{\mathcal{X}}_{k,t}$), direct short-wave ($\mathbf{i}^{\mathcal{X}}_{k,t}$ and $\hat{\mathbf{i}}^{\mathcal{X}}_{k,t}$), and direct long-wave ($\mathbf{r}^{\mathcal{X}}_{k,t}$ and $\hat{\mathbf{r}}^{\mathcal{X}}_{k,t}$).

Standard weather features: atmospheric pressure ($\mathbf{p}^{\mathcal{X}}_{k,t}$ and $\hat{\mathbf{p}}^{\mathcal{X}}_{k,t}$), dew point ($\mathbf{d}^{\mathcal{X}}_{k,t}$ and $\hat{\mathbf{d}}^{\mathcal{X}}_{k,t}$), relative humidity ($\mathbf{h}^{\mathcal{X}}_{k,t}$ and $\hat{\mathbf{h}}^{\mathcal{X}}_{k,t}$), and air temperature ($\mathbf{t}^{\mathcal{X}}_{k,t}$ and $\hat{\mathbf{t}}^{\mathcal{X}}_{k,t}$).

Wind velocity magnitude: 10 m ($\mathbf{w}^{10\mathcal{X}}_{k,t}$ and $\hat{\mathbf{w}}^{10\mathcal{X}}_{k,t}$), 60 m ($\mathbf{w}^{60\mathcal{X}}_{k,t}$ and $\hat{\mathbf{w}}^{60\mathcal{X}}_{k,t}$), 80 m ($\mathbf{w}^{80\mathcal{X}}_{k,t}$ and $\hat{\mathbf{w}}^{80\mathcal{X}}_{k,t}$), 100 m ($\mathbf{w}^{100\mathcal{X}}_{k,t}$ and $\hat{\mathbf{w}}^{100\mathcal{X}}_{k,t}$), and 120 m ($\mathbf{w}^{120\mathcal{X}}_{k,t}$ and $\hat{\mathbf{w}}^{120\mathcal{X}}_{k,t}$).

Apparent temperature features: discomfort index ($\mathbf{s}^{\mathcal{X}}_{k,t}$ and $\hat{\mathbf{s}}^{\mathcal{X}}_{k,t}$), and wind chill ($\mathbf{c}^{\mathcal{X}}_{k,t}$ and $\hat{\mathbf{c}}^{\mathcal{X}}_{k,t}$).

It is possible to combine the mask from different $\mathcal{X}$ energy features, such as solar $\mathcal{S}$ and wind $\mathcal{W}$, so that $\mathbf{x}^{\mathcal{W}}_{k,t} = \{\mathrm{X}_{i,j,k,t} \mid m^{\mathcal{S}}_{i,j} > 0 \vee m^{\mathcal{W}}_{i,j} > 0\}$ with dimensions $\mathbf{x}^{\mathcal{W}}_{k,t} \in \mathbb{R}^{D^{\mathcal{SW}}}$, where $D^{\mathcal{SW}} = \sum_{i,j} \mathbb{I}\left(m^{\mathcal{SW}}_{i,j} > 0\right)$. The predictor variables in the 24-hour post-processing forecast are the energy demand or VRE generation. Solar generation (NP15, SP15, and ZP26) and energy demand have 3 spatial components (PGE, SCE, and SDGE), but wind only has 2 (NP15 and SP16). NP15 and PGE are trading hubs and utilities in northern California, SP15 and SDGE are trading hubs and utilities in southern California, and ZP26 and SCE are trading hubs and utilities serving parts of the central coast and southern California.

Supplementary Note 6

The pattern vectors may include temporal features $z_{k,1}$ (year), $z_{k,2}$ (year-day), $z_{k,3}$ (day-hour), $z_{k,4}$ (weekday), $z_{k,5}$ (weekend), $z_{k,6}$ (Holiday) and $z_{k,7}$ (daylight saving time). We consider the year as a continuously increasing variable from the first year in the time series,

$$z_{k,1} = \mathrm{year} - 2019, \tag{14}$$

to capture the increases in the generation from the cumulative installed capacity and energy demand related to GDP growth.

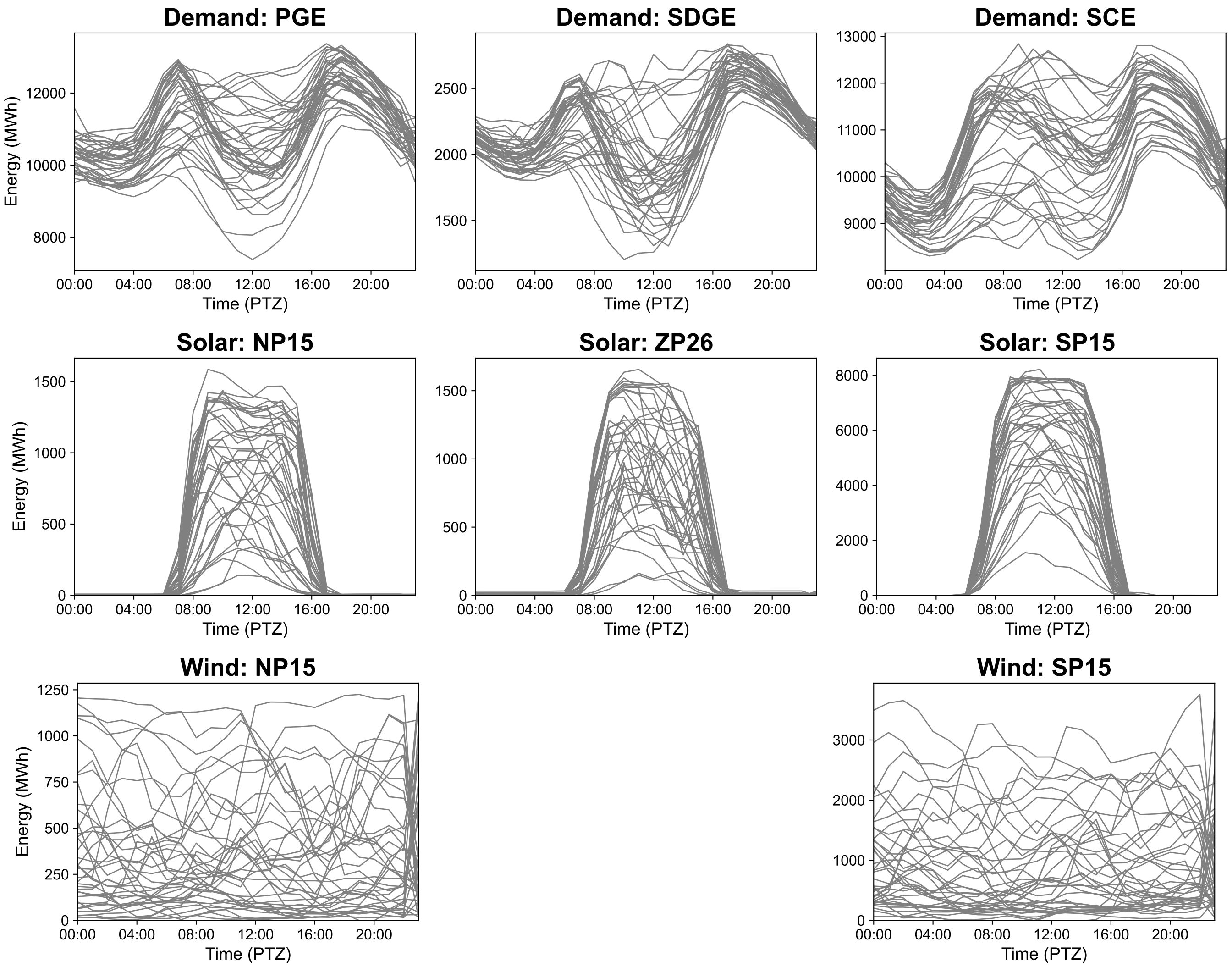

Supplementary Figure 25: Top row panels, hourly energy demand on 39 days first days of 2023 from the largest customer-serving utilities in California (PGE, SDGE, and SCE). Middle row panels, hourly solar energy generation at the three different trading hubs operated by CAISO, NP15 (northern California), SP15 (southern California), and ZP26 (central coast). Bottom row panels, hourly wind energy generation at the two CAISO's hubs trading wind energy, NP15 and SP15.

The cumulative counting in year days count, hours count, and weekday count produces that distance between year days, weekdays, or day hours cannot capture intra-year, intra-week, and intra-day cyclostationary. Cumulative time cannot inform when different day hours (e.g. 23 pm and 1 am) or year days (e.g. Jan 1 and Dec 31) may have similar weather due to climate patterns. The cumulative temporal features are transformed into periodic,

$$
\begin{aligned}
z_{k,2} &= \frac{1}{2} - \frac{1}{2}\cos\left(\frac{2\pi}{365.25}\cdot \text{year day}\right), \\
z_{k,3} &= \frac{1}{2} - \frac{1}{2}\cos\left(\frac{2\pi}{24}\cdot \text{hour}\right), \\
z_{k,4} &= \frac{1}{2} - \frac{1}{2}\cos\left(\frac{2\pi}{7}\cdot \text{weekday}\right).
\end{aligned}
\tag{15}
$$

In addition, it accounts for effects in the electric load when changing to Daylight Saving Time (DST).

$$
\begin{aligned}
z_{k,5} &= \begin{cases} 1 & \text{if weekend} \\ -1 & \text{otherwise} \end{cases} \\
z_{k,6} &= \begin{cases} 1 & \text{if Holiday} \\ -1 & \text{otherwise} \end{cases} \\
z_{k,7} &= \begin{cases} 1 & \text{if DST} \\ -1 & \text{otherwise} \end{cases}
\end{aligned} \tag{16}
$$

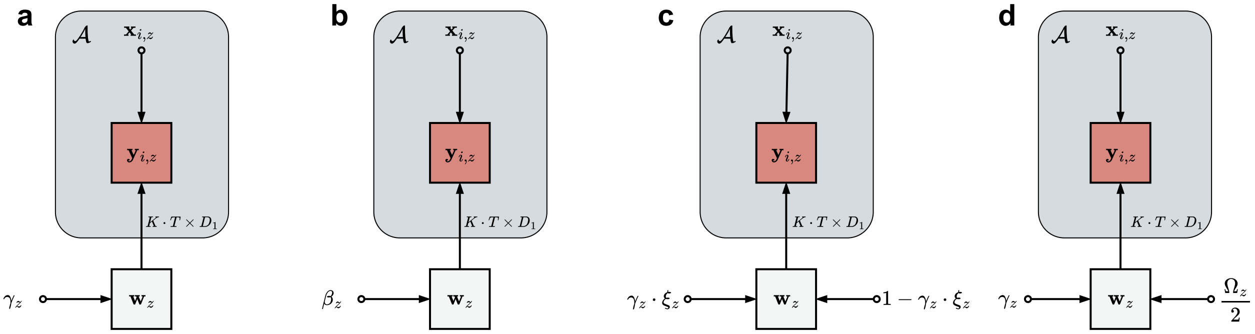

Supplementary Figure 26: Diagram of the different sparse learning methods implemented in this research: lasso (a), orthogonal matching pursuit (b), elastic net (c), and group lasso (d). The large gray box represents the reanalyzed dataset ($\mathcal{A}$). The gray box is the learning parameters, and the variables outside the box are the model hyperparameters. The red box is the independent variable. The arrows depict the information flow.

Supplementary References

[1] Department of Market Monitoring. *2023 Annual Report on Market Issues and Performance*. Tech. rep. California Independent System Operator (CAISO), July 2024.

[2] Department of Market Monitoring. *2020 Annual Report on Market Issues and Performance*. Tech. rep. California Independent System Operator (CAISO), Aug. 2021.

[3] Natural Earth. *Natural Earth vector map data*. Accessed January 2026. 2026. URL: https://www.naturalearthdata.com.

[4] California Energy Commission. *Balancing Authority Areas in California (GIS shapefile)*. Accessed January 2026. 2026. URL: https://www.energy.ca.gov/data-reports/energy-maps-and-spatial-data.

[5] Robert L Winkler. "Scoring rules and the evaluation of probability assessors". In: *Journal of the American Statistical Association* 64.327 (1969), pp. 1073–1078.

[6] Allan H Murphy and Robert L Winkler. "Scoring rules in probability assessment and evaluation". In: *Acta psychologica* 34 (1970), pp. 273–286.

[7] Mathias Blicher Bjerregård, Jan Kloppenborg Møller, and Henrik Madsen. "An introduction to multivariate probabilistic forecast evaluation". In: *Energy and AI* 4 (2021), p. 100058.

[8] Philippe Lauret, Mathieu David, and Pierre Pinson. "Verification of solar irradiance probabilistic forecasts". In: *Solar Energy* 194 (2019), pp. 254–271.

[9] Tilmann Gneiting and Adrian E Raftery. "Strictly proper scoring rules, prediction, and estimation". In: *Journal of the American statistical Association* 102.477 (2007), pp. 359–378.

[10] Michael Scheuerer and Thomas M Hamill. "Variogram-based proper scoring rules for probabilistic forecasts of multivariate quantities". In: *Monthly Weather Review* 143.4 (2015), pp. 1321–1334.

[11] John Shawe-Taylor and Nello Cristianini. *Kernel Methods for Pattern Analysis*. New York, NY, USA: Cambridge University Press, 2004. ISBN: 0521813972.

[12] Ernest W Peterson and Joseph P Hennessey Jr. "On the use of power laws for estimates of wind power potential". In: *Journal of Applied Meteorology and Climatology* 17.3 (1978), pp. 390–394.

[13] Earl Crabill Thom. "The discomfort index". In: *Weatherwise* 12.2 (1959), pp. 57–61.
